# Novel hybrid evolutionary algorithm for bi-objective optimization problems

**DOI:** 10.1038/s41598-023-31123-8

**Published:** 2023-03-15

**Authors:** Omar Dib

**Affiliations:** Wenzhou-Kean University, Wenzhou, China

**Keywords:** Applied mathematics, Computer science

## Abstract

This work considers the Bi-objective Traveling Salesman Problem (BTSP), where two conflicting objectives, the travel time and monetary cost between cities, are minimized. Our purpose is to compute the trade-off solutions that fulfill the problem requirements. We introduce a novel three-Phase Hybrid Evolutionary Algorithm (3PHEA) based on the Lin–Kernighan Heuristic, an improved version of the Non-Dominated Sorting Genetic Algorithm, and Pareto Variable Neighborhood Search, a multi-objective version of VNS. We conduct a comparative study with three existing approaches dedicated to solving BTSP. To assess the performance of algorithms, we consider 20 BTSP instances from the literature of varying degrees of difficulty (e.g., euclidean, random, mixed, etc.) and different sizes ranging from 100 to 1000 cities. We also compute several multi-objective performance indicators, including running time, coverage, hypervolume, epsilon, generational distance, inverted generational distance, spread, and generalized spread. Experimental results and comparative analysis indicate that the proposed three-phase method 3PHEA is significantly superior to existing approaches covering up to 80% of the true Pareto fronts.

## Introduction

The Traveling Salesman Problem (TSP) is one of the most studied combinatorial optimization problems^1^. That is driven by its theoretical significance and applicability in various fields such as microchips and printed circuit board designing^2^, DNA sequencing^3^, and platform allocation^4^. Several routing issues, logistic-based applications, and operations research problems such as task scheduling^5^ and stock cutting^6^ can be modeled as a variant of TSP or involve solving a TSP in one of their critical sub-problems.

The TSP focuses on a traveler seeking the shortest possible distance to visit multiple cities, each only once, and eventually returning to the starting place. The traveler can be a salesman, school bus, autonomous car, drone, or broadly any entity that has to optimize a cost function while visiting multiple places, each only once^7^. In the traditional TSP, the aim is to find the target circuit that has the shortest possible distance among all existing possibilities. However, a single objective configuration does partially characterize real-world standards. Essentially, travelers often tend to consider multiple objectives simultaneously while moving from one place to another, such as monetary cost, travel time, comfort, etc. It is usually not an optimal choice for all travelers to reach the destination in a shorter amount of time at the expense of a non-affordable price. It is, therefore, crucial to allow decision-makers to select the most preferred choice (s) based on their traveling objectives^8^.

Solving TSP turns out to be challenging due to the NP-hard nature of the problem^7^. Therefore, due to high computational time issues, relying on exact algorithms to deal with TSP is not a viable option, especially when the problem’s input size is large. When multiple objectives are concurrently considered, solving a TSP becomes even more challenging and complex, as, in practice, one problem might have many trade-off solutions^9^. Computing those solutions either optimally or approximately and helping users with the intricate decision-making process is the backbone of multi-objective optimization. In practice, multi-objective optimization involves making decisions in the presence of trade-offs between two or more conflicting objectives^10^. Minimizing cost, maximizing quality while buying a product, and maximizing performance while reducing fuel consumption and CO$_2$ vehicle emissions are examples of multi-objective problems^11^. There can be more than three objectives in practice, which is referred to as many-objective optimization^12^.

For nontrivial multi-objective problems, no single solution simultaneously optimizes each objective. A solution is called non-dominated, Pareto optimal, or non-inferior if none of the objectives can be improved without degrading the other objective values. In practice, a (possibly infinite) number of Pareto optimal solutions may exist, all of which are considered equally good. Therefore, the goal in multi-objective optimization is to find a representative set of Pareto optimal solutions and select one or a small subset of solutions that satisfy the subjective preferences of the decision-maker.

To compute the trade-off solutions effectively, there has been a continuous effort to apply approximate approaches such as metaheuristics. Such methods, under careful design, tend to provide high-quality trade-off solutions for multi-objective optimization problems in an acceptable computation time. In this work, we have opted for using multi-objective evolutionary algorithms to solve the underlying bi-objective TSP. We aim to provide decision-makers with a representative set of non-inferior TSP circuits from which they can select the most preferred solution(s). We consider travel time and cost as two conflicting criteria under minimization. We propose a three-phase method named “Three-Phase Hybrid Evolutionary Algorithm” (3PHEA) based on the Lin–Kernighan Heuristic (LKH), an improved version of the Non-Dominated Sorting Genetic Algorithm (NSGA-II) and Pareto Variable Neighborhood Search (PVNS) a multi-objective version of VNS. Our objective is to adapt and combine the characteristics of different metaheuristics to obtain higher quality trade-off solutions than existing solutions for the studied problem.

The contributions of this work are as follows:

- improve solving the bi-objective TSP by proposing a three-phase method (3PHEA) based on the Lin–Kernighan Heuristic (LKH), an enhanced version of the Non-Dominated Sorting Genetic Algorithm (NSGA-II), and Pareto Variable Neighborhood Search (PVNS),
- compare the proposed approach (3PHEA) with existing methods from state of the art to illustrate its similarities, differences, and effectiveness,
- study several multi-objective performance indicators to assess and compare the proposed algorithms, including coverage, generational distance, inverse generational distance, hypervolume, epsilon, spread, generalized spread, and time complexity metrics,
- solve twenty bi-objective real-world instances ranging from 100 to 1000 cities and conduct detailed comparative and assessment studies.

The rest of the paper is organized as follows. In “Literature review”, we present state-of-the-art related to the studied problem and applied algorithms. In “Problem definition”, we formulate the multi-objective TSP problem. “Proposed algorithms” presents the proposed method (3PHEA). We discuss the experimental studies, including the experimental setup, test instances, performance metrics, and results analysis in “Experimental study”. Finally, we discuss the limitations of the proposed algorithms in “Discussions” and highlight conclusions and future works in “Conclusions”.

## Literature review

TSP is one of the most popular NP-hard problems in classical combinatorial optimization. Due to its simple formulation, challenging computation, and wide applications, TSP has received significant attention from theoreticians and practitioners^13^. There are currently many optimal and approximate algorithms to solve TSP or one of its variants. Metaheuristics such as the population-based Genetic Algorithm (GA), the single solution-based Tabu Search (TS), and Variable Neighborhood Search (VNS) are among the methods that have been utilized to handle basic and complex versions of TSP^14^. Such approaches have shown remarkable performance in practice when solving optimization problems under various settings such as deterministic, stochastic, single objective, multiple objectives, time-dependent, etc. Adding to their computation efficiency, metaheuristics tend to provide robust solutions even for large-scale optimization problems^15^.

Among metaheuristics, GAs have been applied to solve optimization problems in various sectors. For instance, in^16^, the authors proposed a GA-based scheme for planning and managing aircraft maintenance. Authors of^17^ proposed a spatial GIS-based GA for route optimization of waste collection. Based on those works, GA’s performance was practically convincing for real-world use cases. Another prominent algorithm that has been considered for solving large-scale combinatorial optimization problems is VNS. VNS is mainly popular for its capacity to overcome local minima using dynamic neighborhood strategies. Recently, VNS was used by the authors of^18^ to introduce a hybrid adaptive large neighborhood search algorithm for the capacitated routing problem. Authors of^19^ also recently proposed a novel VNS with tabu shaking for a class of multi-depot vehicle routing problems. In both works, VNS performance was remarkably superior to conventional metaheuristics.

To enhance the ability of metaheuristics to efficiently explore an objective space, recent works have combined several methods for solving a given problem^20^. Those works claim that combining several metaheuristics improves the exploration and exploitation and thereby performs better in practice. For example, in^21–23^, the authors computed near-optimal multimodal routes in a reasonable amount of computation time in large-scale transportation networks by combining VNS and GA. And in^24^, authors proposed a hybrid algorithm combining several simulated annealing strategies, which significantly enhanced the algorithm’s convergence and accuracy. Hybrid methods are indeed efficient. However, their design is not an easy task as it might cause solving redundant tasks and thereby lead to additional computational overhead. The above-discussed metaheuristics have shown noticeable performance when solving the traditional single-objective version of TSP. However, in real life, travelers often need to simultaneously consider several aspects during their trips, such as travel expenses, traffic conditions, and comfort^25^.

Multi-objective optimization has been receiving significant attention, and many algorithms have been proposed to optimize under the presence of conflicting objectives. For example, Deb et al.^26^ early introduced a multi-objective evolutionary algorithm, the Non-dominated Sorting Genetic Algorithm II (NSGA-II), that balances the quality of non-dominated solutions and their diversity via non-domination sort and crowding distance mechanisms. NSGA-II and its variants have been effectively applied for solving multi-objective problems in various fields of application. For example, in^27^, the authors adapted NSGA-II for fault diagnosis in a power system. In addition, in^28^, authors proposed a two-level resource scheduling model and designed a resource scheduling scheme among fog nodes in the same fog cluster based on NSGA-II. Their MATLAB simulation results showed that NSGA-II effectively reduces the service latency and improves the stability of the task execution.

Other well-known multi-objective approaches involve decomposition-based algorithms that transform a multi-objective problem into a set of single-objective problems using scalarizing functions. The resulting single-objective sub-problems are then solved simultaneously. Several algorithms are proposed under this category, such as multi-objective genetic local search, cellular multi-objective genetic algorithm, and multi-objective evolutionary algorithm based on decomposition. Recent applications of those algorithms can be found here, respectively^29–31^. Alternatively, there has been a consistent effort to use multi-objective performance indicators to guide evolutionary algorithms. Those algorithms attempt to find the best subset of trade-off solutions based on a performance indicator. Many variants can be looked at in this regard, such as indicator-based-selection evolutionary algorithm, s-metric selection evolutionary multi-objective optimization algorithm, fast hypervolume multi-objective evolutionary algorithm, and many-objective metaheuristic based on R2 indicator. Recent studies applying those algorithms can be found here, respectively^32–35^. Novel evolutionary approaches have been also proposed for multi-objective optimization. For example, the authors of^36^ introduced a multi-objective variant of chemical reaction optimization, which is a metaheuristic inspired by chemical reactions launched during collisions. The method uses a new quasi-linear average time complexity quick nondominated sorting algorithm to improve the computational cost. The new method was applied on DTLZ multi-objective test suite^37^ and showed promising performance. However, the authors indicate in the conclusions of their paper that the results cannot be automatically generalized to real word problems. Other new evolutionary algorithms, such as^38,39^ have been introduced and applied on the mathematically generated test instances ZDT test suite^40^, and DTLZ test suite^37^. Similar to^36^, those approaches are not guaranteed to perform well or might not be suitable for real-world problems, such as the multi-objective TSP, which involves conflicting objectives.

Focusing on multiobjective TSP problems, several attempts have been made to use metaheuristics to compute or approximate the optimal Pareto front. For example, authors of^41^ presented a method called Two-Phase Pareto Local Search (2PPLS) to find a good approximation of the efficient set of the bi-objective TSP. In the first phase of the method, the authors use the Concorde TSP solver^42^ to generate an initial population composed of a good approximation of the extremely supported efficient solutions. Then, in the second phase, they apply a Pareto Local Search method to each solution of the initial population to approximate the non-supported efficient solutions. Their experimental results showed improvements compared to^43^ but were not assessed against the true Optimal Pareto fronts. Their instances and results were made public and can be found here^44^. 2PPLS, initially proposed for bi-objective problems, was extended to handle many objectives of euclidean TSP in^45^. 2PPLS was also applied to solve bi-objective problems in different domains, such as bi-objective pollution-routing problem^46^ and device allocation in the distributed integrated modular avionics^47^.

In 2014, Florios and Mavrotas^48^ presented AUGMECON2, an Augmented Epsilon Constraint Method to generate the exact Pareto set for multi-objective integer programming problems. AUGMECON2 was used to generate the exact Pareto fronts of many instances of two popular multi-objective problems, namely, the multi-Objective TSP and the multi-Objective set covering problem. Despite its high computational time, AUGMECON2 was effectively used to compute the Pareto fronts of Lust’s multi-objective TSP instances^41^ and Paquete’s instances^43^. Interestingly, all results were made public and can be accessed from here^49^.

Population-based methods were also proposed for solving multi-objective TSP. For example, the authors of^50^ compared single and multiple objective evolutionary algorithms to solve the bi-objective TSP and knapsack problems. Their results show that multiobjective algorithms are more effective than single-objective algorithms even though they are executed repeatedly. They also show that multiobjective algorithms exhibit better behavior when dealing with large instances or instances with strongly correlated objectives. In^51^, authors proposed a new approach based on NSGA-II, SPEA2, and decomposition features for solving several instances of the bi-objective TSP problem. Their experimental results demonstrate the effectiveness of their method. However, the time complexity is omitted from the study. In^52^, authors developed a two-stage evolutionary algorithm (TSEA) for the multiobjective TSP. The first stage involves using a hybrid local search evolutionary algorithm (HLS-EA) which incorporates the 2-opt local search in NSGA-II to solve the individual objectives of multiobjective TSP. These individual single-objective solutions representing the corner solutions of the Pareto optimal front are used in the second stage as seed solutions in seeded HLS-EA (SHLS-EA) for solving the corresponding multiobjective ETSP. The algorithm showed superior performance compared with several variants of GA, even though no relevant empirical details were given. More precisely, the quality of obtained fronts was not compared against the true Pareto fronts for the various considered instances making the assessment of the method relatively biased.

Differently, in^8^, authors introduced a rule-based Artificial Bee Colony (ABC) algorithm in which the fitness is determined based on a set of rules following the dominance property. The lexicographical rules are used as the core of a roulette wheel selection process. To preserve diversity, a crowding distance operator is used. For performance assessment, authors solved several TSP lib instances ranging from 100 to 300 cities. The authors claim that the results show that the proposed approach is efficient enough to solve multiobjective TSP. Nonetheless, no comparison with an optimal algorithm was performed, making assessing the obtained Pareto fronts unfair. In addition, the time indicator was not included in the experimental assessment. Furthermore, authors of^53^ addressed the multiobjective TSP using an evolutionary-based algorithm with random immigrants. The latter was used to increase the population’s diversity and allow for a better exploration of a larger area of the search space. Interestingly, authors indicated and experimentally showed that introducing random immigrants while using local search procedures with an evolutionary algorithm incurs a certain overhead which is especially significant in combinatorial optimization. That is, relying on blind operators might increase the diversity but leads to a severe increase in computational time. The authors also found that decreasing the number of immigrants with time, an idea similar to simulated annealing, improves multiobjective optimization results in terms of both the hypervolume and the IGD indicators.

Owing to the relevance of the multiobjective TSP and to fill the gap in analyzing existing approaches, proposing efficient hybrid algorithms, and studying their empirical behaviors from a time and quality points of view, we introduce 3PHEA, a novel three phases hybrid evolutionary approach for solving the bi-objective TSP. Our approach proceeds with an initial population generated based on the Lin–Kernighan Heuristic, then improved using a Hybrid Non-Dominated Sorting Genetic Algorithm, and finally consolidated using a Pareto Variable Neighborhood Search.

## Problem definition

**Figure 1:**
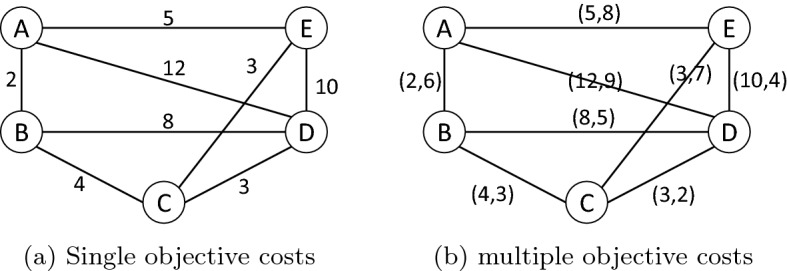
Illustration of single and bi-objective TSP graphs.

TSP is defined by a set of cities and the distances between each city pair. The problem is to find a circuit that goes through each city once and that ends where it starts. Consider the following set of cities shown in Fig. 1a.

The problem consists of finding a minimal path passing through all vertices once. For example, the path $P_1 = {A, B, C, D, E, A}$ and the path $P_2 = {A, B, C, E, D, A}$ pass through all vertices, but $P_1$ has a total length of 24, and $P_2$ length is 31. As a multi-objective problem, other notions, such as time and cost, can be considered, as is shown in Fig. 1b. $P_1$ has a total cost of (24, 23) and $P_2$ cost vector is (31, 29). The mathematical model of the TSP can be expressed as:

1 $$\begin{aligned}&\text {min} \qquad{} & {} F(x)=\left( F_{1}(x), F_{2}(x), \ldots F_{m}(x)\right) \end{aligned}$$

2 $$\begin{aligned}&\text {where} \qquad{} & {} F_{1}(x)=\sum _{i=1}^{n-1} C^{1}\left( x_{i}, x_{i+1}\right) +C^{1}\left( x_{n}, x_{1}\right) \end{aligned}$$

3 $$\begin{aligned}{} & {} {}&\ldots , \end{aligned}$$

4 $$\begin{aligned}{} & {} {}&F_{m}(x)=\sum _{i=1}^{n-1} C^{m}\left( x_{i}, x_{i+1}\right) +C^{m}\left( x_{n}, x_{1}\right) \end{aligned}$$

5 $$\begin{aligned}&\text {s.t.} \qquad{} & {} \sum _{j} x_{i j}=1, \quad j \ne i \text{ for } \text{ each } i \in V \end{aligned}$$

6 $$\begin{aligned}{} & {} {}&\sum _{i} x_{i j}=1, \quad i \ne j \text{ for } \text{ each } j \in V \end{aligned}$$

where *m* is the number of objectives, *n* is the number of cities, $C^m$ is the traveling measure, *x* is the decision vector, and $X \in R^n$ is the *n*-dimensional decision space. *F*(*x*) represents the objective function, and $F \in R^m$, the *m*-dimensional objective space. The single-objective problem is typically studied in decision space, whereas multi-objective optimization is mostly studied in objective space. The image of a solution in the objective space is a point, $\textrm{F}=\left[ \textrm{F}_{1}, \mathrm {~F}_{2}, \ldots \ldots \textrm{F}_{\textrm{m}}\right]$. A point $\textrm{F}$ is attainable if there exists a solution $x \in X$ such that $F=F(x)$. The set of all attainable points is denoted as $\textrm{F}$. The ideal objective vector $\textrm{F}^{*}$ is defined as $\textrm{F}^{*}=\left[ {\text {opt}} \textrm{F}_{1}(\textrm{x}), {\text {opt}} \textrm{F}_{2}(\textrm{x}), \ldots , \right.$ opt $\left. F_{m}(x)\right]$, which is obtained by optimizing each of the objectives individually.

### Definition 1

Any solution *x* that satisfies all constraints and variable bounds is denoted as a feasible solution.

### Definition 2

A vector $a=(a_1,\ldots ,a_m)$ dominates another vector $b=(b_1,\ldots ,b_m)$, ($a \prec b$), if and only if, $a_k \le b_k \; \forall k \in \{1,\ldots ,m\} \wedge \exists \; k \in \{1,\ldots ,m\}: a_k<b_k$

### Definition 3

A vector $a=(a_1,\ldots ,a_m)$ strictly dominates another vector $b=(b_1,\ldots ,b_m)$, ($a < b$), if and only if, $a_k < b_k \; \forall k \in \{1,\ldots ,m\}$

### Definition 4

A vector $a=(a_1,\ldots ,a_m)$ weakly dominates another vector $b=(b_1,\ldots ,b_m)$, ($a \le b$), if and only if, $a_k \le b_k \; \forall k \in \{1,\ldots ,m\}$

Based on definitions 2, 3, and 4, we can write:

7 $$\begin{aligned} (a < b) \Rightarrow (a \prec b) \Rightarrow (a \le b) \end{aligned}$$

### Definition 5

A feasible vector $x^{0} \in X \quad$ (X is the feasible region) yields a non-dominated solution, if and only if, there is no other feasible vector $\textrm{x} \in \textrm{X}$ such that,

8 $$\begin{aligned} \sum _{i=1}^{m} \sum _{j=1}^{n} c_{i j}^{k} x_{i j} \le \sum _{i=1}^{m} \sum _{j=1}^{n} c_{i j}^{k} x_{i j}^{0}, \text{ for } \text{ all } k, and \end{aligned}$$

9 $$\begin{aligned} \begin{aligned}{}&\sum _{i=1}^{m} \sum _{j=1}^{n} c_{i j}^{k} x_{i j}<\sum _{i=1}^{m} \sum _{j=1}^{n} c_{i j}^{k} x_{i j}^{0}, \\ \text{ for } \text{ some } k \text{, }&\textrm{k}=1,2, \ldots \ldots , \textrm{m} \end{aligned} \end{aligned}$$

### Definition 6

A point $x^{0} \in X$ is efficient if and only if there does not exist another $\textrm{x} \in \textrm{X}$ such that,

10 $$\begin{aligned} \sum _{i=1}^{m} \sum _{j=1}^{n} c_{i j}^{k} x_{i j} \le \sum _{i=1}^{m} \sum _{j=1}^{n} c_{i j}^{k} x_{i j}^{0}, \text{ for } \text{ all } k \end{aligned}$$

11 $$\begin{aligned} \sum _{i=1}^{m} \sum _{j=1}^{n} c_{i j}^{k} x_{i j} \ne \sum _{i=1}^{m} \sum _{j=1}^{n} c_{i j}^{k} x_{i j}^{0}, \text{ for } \text{ some } k \end{aligned}$$

### Definition 7

A feasible vector $\textrm{x}^{*} \in \textrm{X}$ is called a compromise solution if $x^{*} \in E$ and $F\left( x^{*}\right) \le \Lambda _{x \in X} F(x)$, where $\Lambda$ stands for “minimum” and $\textrm{E}$ is the set of efficient solutions.

### Definition 8

If the compromise solution satisfies the decision maker’s preferences, then the solution is called the preferred compromise solution.

### Definition 9

The multi-objective problem can be compounded into a single objective optimization problem by a linear combination of the multiple objectives with weights, i.e., form a composite objective function as the weighted sum of the objectives, where the objective is to minimize the linear combination of the multiple objectives with weights which minimize a positively weighted convex sum of the objective.

12 $$\begin{aligned}&\text {min}{} & {} \sum _{i=1}^{m} \alpha _{i} F_{i}(x) \quad and \quad \sum _{i=1}^{m} \alpha _{i}=1, \end{aligned}$$

13 $$\begin{aligned}&\text {where}{} & {} \alpha _{i}>0, \text { weightage for the } \textrm{i}{ \text{ th } } \text{ objective } \text{ and } x \in X. \end{aligned}$$

### Definition 10

Supported efficient solutions are all the optimal solutions that can be obtained by solving the corresponding weighted sum single objective problems for some vector $\lambda > 0$. The image in the objective space of the supported efficient vectors is located on the lower left boundary of the convex hull of *F*.

### Definition 11

Non-supported efficient solutions are all the efficient solutions that are not optimal solutions for any weighted sum single-objective problem. The non-supported points in the objective space are located in the interior part of the convex hull of *F*.

## Proposed algorithms

**Figure 2:**
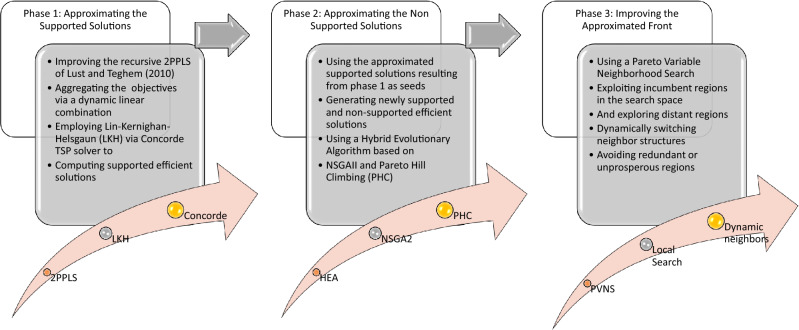
Illustration of the proposed method.

We aim in this paper to solve the bi-objective TSP via a novel three-phase hybrid evolutionary algorithm (3PHEA). In the first phase, a set of supported non-dominated solutions are computed, followed by an improvement phase based on a refined version of NSGAII. Lastly, the third phase exploits the improved solutions by a Pareto VNS. The three phases are summarized in Fig. 2; they are complementary and detailed in the following subsections.

### Phase 1: approximating the supported solutions

The first phase of (3PHEA) is an improved version of the method of Lust and Teghem (2010)^41^. This method has shown remarkable performance in solving bi-objective TSP instances as it intelligently computes the supported efficient solutions based on the most popular solver for single-objective TSP^42^. To the best of our knowledge, the latter solver has been the most performant tool for solving single-objective TSP instances. The method consists of generating all weights combinations that make it feasible to obtain a minimal set of supported efficient solutions. For each generated weight set, a linear aggregation of the objectives is carried out, and the resulting single-objective problem is solved by an exact or heuristic method. In this work, as the use of an exact method to solve single-objective problems is extremely time-consuming, we have heuristically adapted the method of Lin–Kernighan–Helsgaun (LKH)^54^. As a result, the obtained solutions are not necessarily efficient, nor supported efficient but constitute a set of solutions that are very close to a minimal complete set of extreme supported efficient solutions.

**Figure 3:**
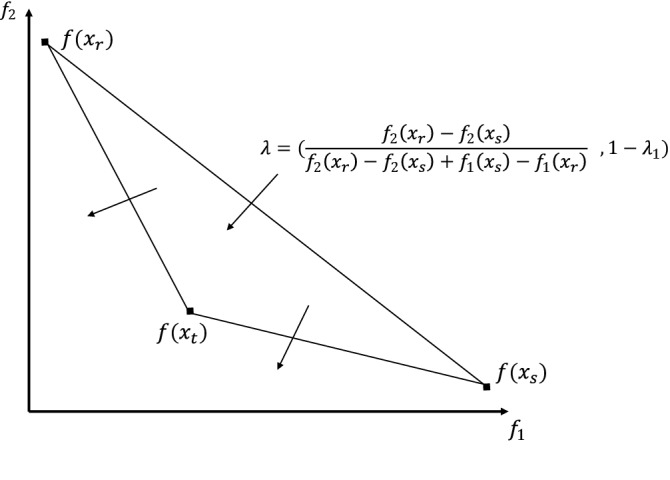
Illustration of Phase 1.

To find an approximation of the extreme supported efficient solutions, we follow the steps of Algorithm 1 called “Phase1: Heuristic”, which uses the Algorithm 2 called “Phase1: Recursion”. Initially, the set ${\hat{S}}$ containing the supported efficient solutions is initialized with two lexicographic solutions corresponding to ${\text {lexmin}}_{x \in X}\left( f_{1}(x), f_{2}(x)\right)$ and ${\text {lexmin}}_{x \in X}\left( f_{2}(x), f_{1}(x)\right)$, respectively. To compute a solution for the weighted sum resulting problem, we employ the LKH heuristic implemented within the Concorde TSP solver^42^; the implementation of this heuristic can be found here^55^. The TSP is first solved by only considering the first objective; the weight vector is thus equal to (1, 0), and the resulting solution is named $x_1$. Similarly, $x_2$ is computed by using the weight vector (0, 1) and its corresponding cost matrix $C^2$. After computing $x_1$ and $x_2$, the dichotomic scheme presented in Algorithm 2 is started. This recursive process is initialized with $x_1$ and $x_2$ $(x_r=x_1 and x_s = x_2)$; subsequently, a single-objective problem is solved with a weight vector representing the normal to the line through $(f(x_r), f(x_s))$. This corresponds to the following $\lambda$ vector: ($\lambda _{1}=\frac{f_{2}\left( x_{r}\right) -f_{2}\left( x_{s}\right) }{f_{2}\left( x_{r}\right) -f_{2}\left( x_{s}\right) +f_{1}\left( x_{s}\right) -f_{1}\left( x_{r}\right) }$ ,$\lambda _{2}=1-\lambda _{1}$). The solution to this problem is named $x_t$ (see Fig. 3). Since the values taken by the weight sets can be very large, we normalize the weight sets such that $\lambda _1 + \lambda _2 = 1$. Accordingly, we round the coefficients of the matrix $C^\lambda$ to the nearest integer value. The resulting solution $x_t$ is added to the approximation set ${\hat{S}}$ via the *addSolution* procedure presented in Algorithm 4.

The procedure *addSolution* (see Algorithm 4) takes an input solution *s* and a list $X_E$ of potentially efficient solutions. To add *s* to $X_E$, the latter is updated such that all solutions dominated by *s* are removed from $X_E$. If a solution *x* from $X_E$ weakly dominates *s* (i.e., $F(x) \le F(s)$), the procedure stops and returns false. Contrarily, true is returned if *s* is added to $X_E$ and all dominated solutions by *s* are removed from $X_E$.

Regarding the stopping criterion of phase 1, when a new solution is computed, the recursion procedure is only called if the solution is located within or to the left of the rectangle formed by the solutions $x_r$ and $x_s$ plus the local nadir and ideal points formed by these two solutions. Since a heuristic method is employed, the solution $x_t$ can also be located outside the defined region. However, executing the procedure from those solutions would reduce the chances of finding new supported or nearly supported efficient solutions.
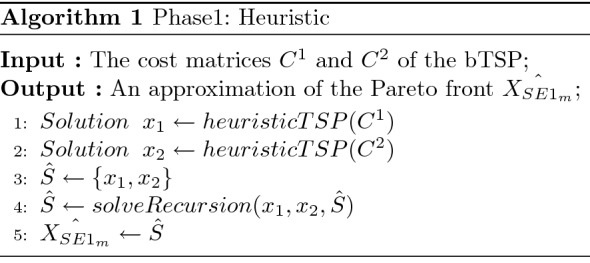

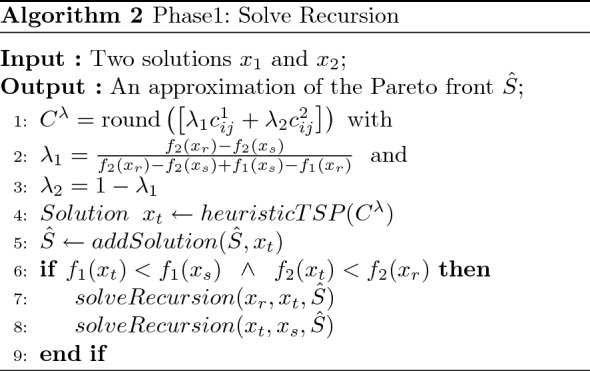


### Phase 2: approximating the non-supported solutions

Phase 2 of the 3PHEA method approximates the set of non-supported efficient solutions. That is crucial as most of the Pareto fronts of real-world multi-objective problems are not entirely convex^11^. In this phase, we employ a hybrid evolutionary algorithm (HEA) based on a non-dominated sorting genetic algorithm (NSGAII) and Pareto Hill Climbing (PHC). NSGAII and PHC have been selected among other heuristic approaches due to their noticeable performance in handling complex objective spaces that encompass convex, and concave regions^26^. In addition, many papers such as^21,23^ have empirically demonstrated the significant results and supreme performance of combining those two methods. The hybridization technique leads to unique exploitation and exploration features, which improve the ability to handle complex Pareto fronts. Nonetheless, other evolutionary algorithms can also be used as alternatives. HEA uses the approximated supported solutions resulting from phase 1 as seeds aiming to generate newly supported and non-supported efficient solutions. The workflow of Phase 2 is presented in Algorithm 3.
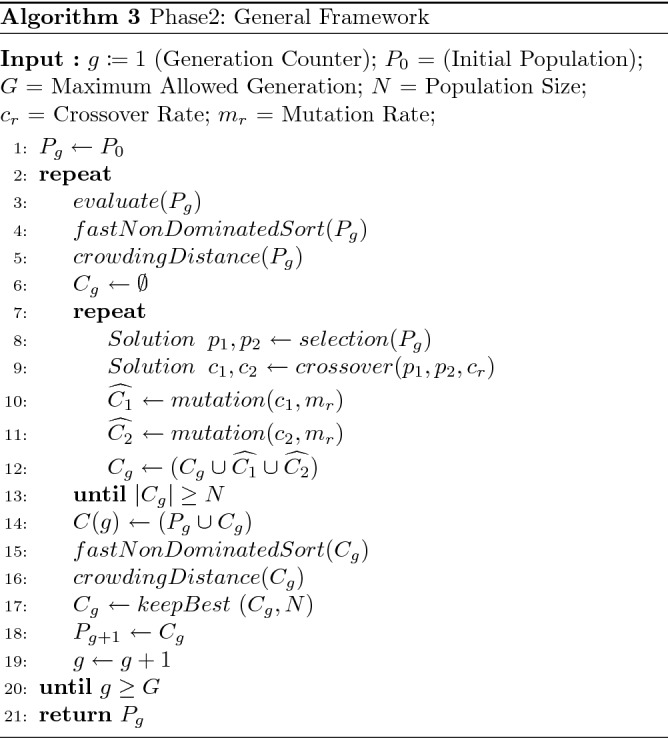


HEA takes as input: an initial population $P_0$ initialized with the set ${\hat{S}}$ resulting from phase 1; a maximum number of allowed generations *G*; the population size *N* =; and $c_r,m_r$ the crossover, and mutation rate, respectively. After evaluating the first population with respect to each objective, individuals are sorted according to their non-domination rank, followed by crowding distance. More specifically, non-dominated solutions in the population are assigned a rank equal to 1; the lower the rank of a solution *s* is, the more *s* is fit and close to the optimal Pareto front. Therefore, classifying solutions based on their non-domination rank is crucial for the parent selection operator. Solutions with a lower rank will be assigned a higher reproduction probability as they are closer to the Pareto optimal front than solutions with a higher rank. Since the rank indicator only accounts for the convergence toward the optimal Pareto front, a crowding distance is used to handle the diversity of the obtained Pareto front. That is, for every solution *s* in the population $P_g$, the crowding distance is computed. Consequently, solutions with a higher crowding distance will be assigned a higher reproduction probability during the parent selection operator. Interestingly, combining the rank and crowding distance in the evolution process helps the algorithm produce well-distributed, non-dominated solutions that are as close as possible to the optimal Pareto front. Intriguingly, the computational complexity of the non-dominated ranking procedure is $O(M*N^2)$, where *M* is the number of objectives and *N* is the population size. Similarly, the crowding distance measure for preserving diversity has a computational complexity of $O(M*NlogN)$.

Following the evolution, selecting two fit individuals is crucial for the crossover and mutation operators to produce better-quality offspring. A tournament selection is used in this algorithm as a selection mechanism. In contrast to single-objective problems, deciding which solution is better is not straightforward when considering multiple objectives. To handle that, two solutions are first compared based on their non-domination rank; a solution with a lower rank is always preferred over a solution with a higher rank. If two solutions have the same rank, the solution with the higher crowding distance is selected. Resulting of the selection operation, two fit individuals are obtained in terms of their closeness to the Pareto optimal front and their distribution.

The Partially Mapped Crossover (PMX) operator is used in this work, although other advanced mechanisms can be applied^56^. This operation is performed by randomly selecting two crossover points that break the two parents $p_1$ and $p_2$ into three sections ($S_1$, $S_2$, and $S_3$). $S_1$ and $S_3$ the sequences of $p_1$ are copied to the child $c_1$, the sequence $S_2$ of $c_1$ is formed by the genes of $p_2$, beginning with the start of its part $S_2$ and leaping the genes that are already established. It is worth mentioning that following this strategy, offspring will always be feasible solutions.

After crossing over two solutions, new routes of better quality and diverse variables are likely to be generated compared to their parents. Therefore, applying a metaheuristic to the offspring will potentially enhance the paths’ qualities. To do so, a multi-objective version of Hill Climbing (HC-MO) presented in Algorithm 5 is used as an alternative to the traditional blind mutation operator. HC-MO proceeds with an initial solution $s_0$, a maximum number of iterations $T_{max}$, and a neighborhood function $N_{k}(x)$. Contrary to traditional hill climbing that repeatedly moves to one (first, last or random) non-dominated neighbor, HC-MO stores the list of non-dominated neighbor solutions. Identifying those non-dominated solutions is done using the fast non-dominated sort procedure of NSGAII. This method can be seen as a growing tree of non-dominated solutions. The tree is developed first from different paths to highlight as many non-dominated solutions from the list of neighbors, then trimmed using the “addSolution” procedure (see Algorithm 4) to keep the best non-dominated solutions. Doing so is a non-trivial step toward extracting relevant solutions from the initial solution $s_0$. $T_{max}$ defines the depth of the tree search; arguably, $T_{max}$ impacts the number of non-dominated solutions and the computational time of HC-MO.
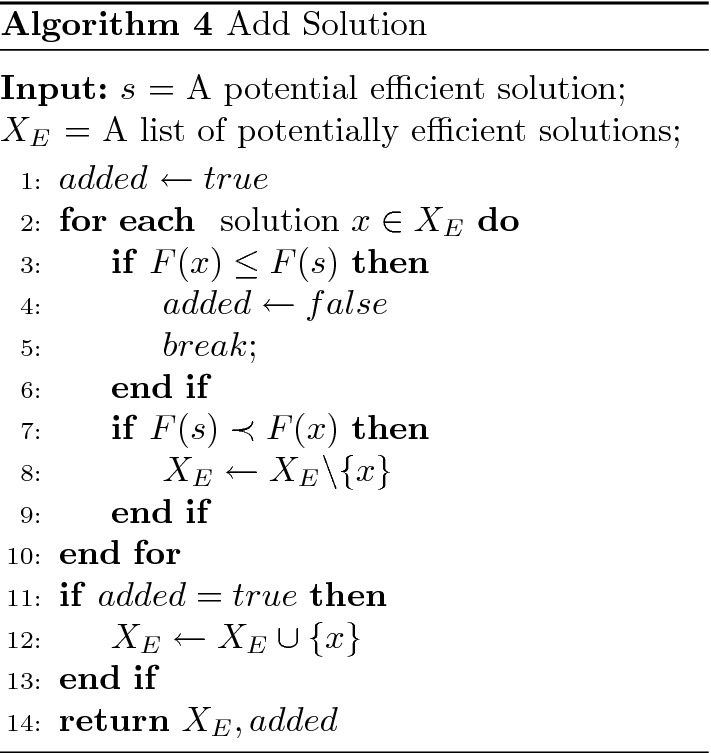

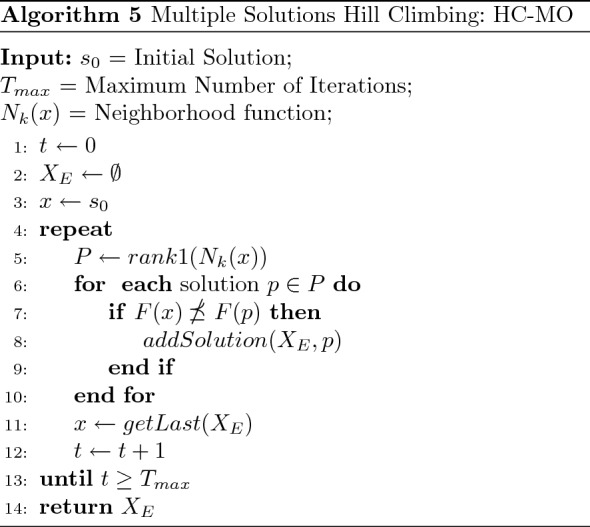


Resulting of the crossover and mutation operations, a new population $C_g$ is produced. To define the population of the next generation, $C_g$ is updated such that it contains the best *N* individuals from the parents and offsprings populations. Deciding whether an individual is better than another follows the domination rank and crowding distance rules. Solutions with the lowest ranks are copied to the new population until the population size is met. If solutions have the same rank, the decision is based on the crowding distance value.

### Phase 3: improving the approximated front

Phases 1 and 2 of 3PHEA approximate the supported and non-supported efficient solutions of the Pareto front, respectively. The resulting set, however, is not totally exploited despite its diversity and quality. To improve the quality of the output population $P_g$ of HEA, we employ a Pareto Variable Neighborhood Search (PVNS) presented in Algorithm 6. This method has been a promising candidate for large and complex multi-objective optimization problems^57^. In addition, one of the features of VNS is its ability to handle local minima by dynamically changing the neighborhood structure. Hence, it strengthens the exploration capacity of 3PHEA and consolidates its exploitation capability thanks to its dynamic local search. Nonetheless, other meta-heuristics such as the Pareto Tabu search^58^, or simulated annealing can be promising candidates for this phase. PVNS takes as input an initial population $P_0$; a neighborhood function $N_{k}(x)$ for each $k \in {\mathbb {Z}}: k_{min} \le k \le k_{max}$; and two variables $k_{min}$ and $k_{max}$ for the index of the first and last neighborhood structure. PVNS has better exploitation and exploration abilities compared to traditional hill climbing as it considers distant regions in the objective space via multiple neighborhood transformations dynamically enforced.

**Figure 4:**
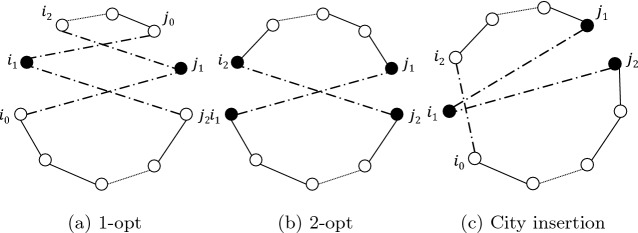
Illustration of neighborhood structures.

PVNS starts with $P_0$, a set of potentially efficient solutions resulting from phase 2. As in a classical VNS, the index *k* of the neighborhood structure is initialized with $k_{min} (k = k_{min})$. Three populations are used: $P_e$ to keep track of the efficient solutions; *P*, the current population containing the solutions to be considered for the search phase of PVNS; and $P_a$, an intermediate population used as an auxiliary set. For each solution *s*, we also add a variable to denote the neighbor functions executed over *s*. For example, executing *setK*(*s*, *n*) means that all neighbor structures from $N_{1}(s)$ to $N_{n-1}(s)$ have already been explored; note that all solutions are initialized with *setK*(*s*, 1) to indicate that for each solution non of the neighbor structures has been explored yet. This function is vital to avoid exploring the same neighborhood of a solution multiple times. Next, searching for new non-dominated solutions is started by exploring all the non-dominated solutions *y* of each solution *x* of *P*. If a neighbor *y* is not weakly dominated by the current solution *x* (i.e., $F(x) \npreceq F(y)$), *y* is added to the efficient solutions $P_e$ via the procedure *addSolution* (see Algorithm 4). If the procedure *addSolution* return *true* (i.e., *y* is not weakly dominated by any solution $x \in P_e$), *y* is added to the intermediate population $P_a$ via *addSolution* and its neighbors variable to initialized to $k_{min}$ using $setK(y,k_{min})$. After exploring all non-dominated neighbor solutions of *P* with respect to the current neighbor transformation $N_k$ and adding them to the intermediate population $P_a$, *k* is set to $k_{min}$ if $P_a$ is not empty. Conversely, *k* is incremented as the non-dominated solutions can be found based on the current neighbor structure. After incrementing *k*, the neighbor variable of the current solutions of $P_e$ that have been explored by $N_{k-1}$ is set to *k* so that they do not get explored multiple times by the same structure. The loop is restarted until all neighbor solutions with respect to all neighbor structure $N_k$ are weakly dominated by one solution from $P_e$ (i.e.,$|P |=0$). In this work, four neighbor structures (i.e., $1 \le k \le 4$), $\{1,2,3\}-OPT$ and city insertion, are applied in PVNS (see Fig. 4). For the implementation details of $\{2,3\}-OPT$, the reader can refer to^59^.
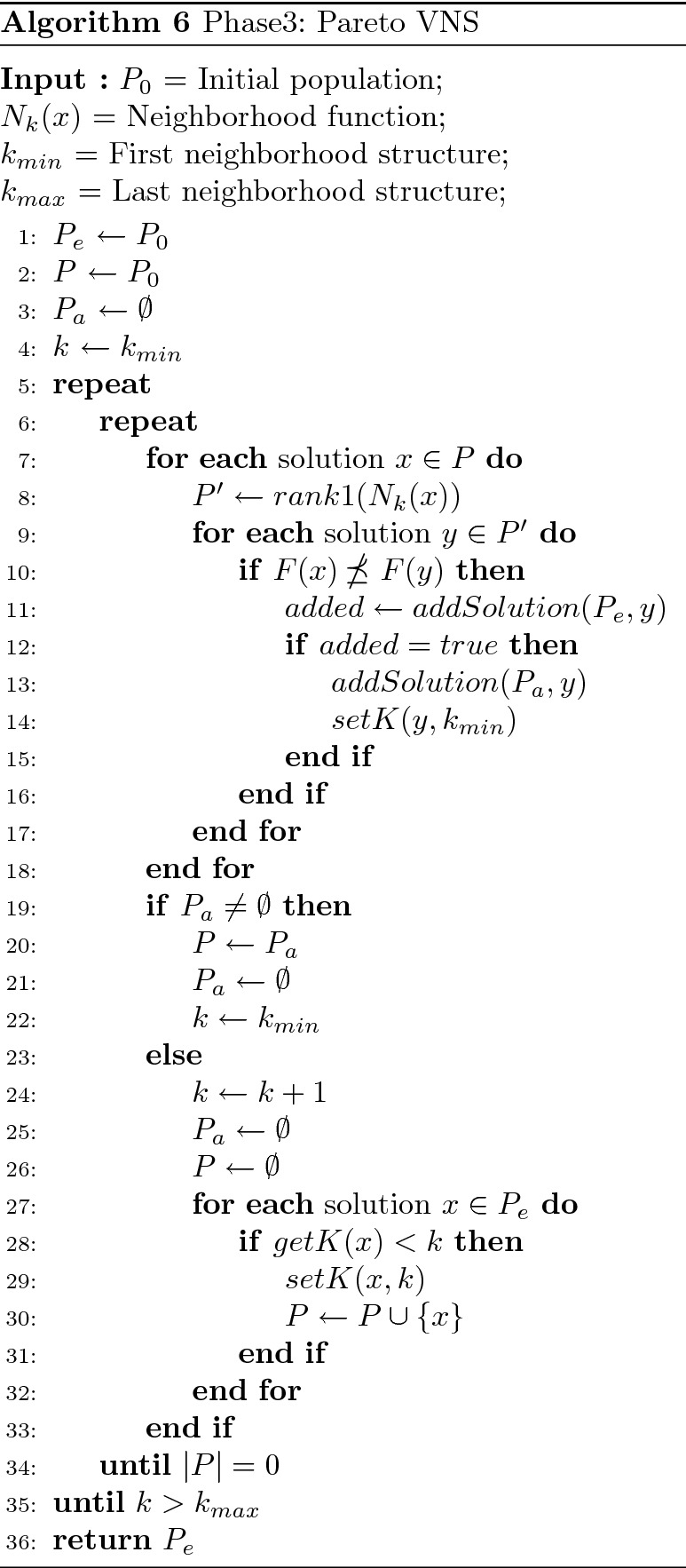

## Experimental study

This section describes the experimental setup, test instances, multi-objective performance indicators, and the analysis of results of 3PHEA and existing algorithms.

### Experimental setup

To assess the performance of the proposed 3PHEA, 20 bi-objective TSP data instances ranging from 100 to 1000 cities (see ***“Test instances5.2”) have been solved. A comparative study was also conducted against existing algorithms with respect to several multi-objective performance indicators. All the algorithms are developed in the Java programming language. Simulations were conducted on a personal computer having the following properties: *Processor*) Intel(R) Core(TM) i7-10850H CPU @ 2.70 GHz, $Installed\;Ram)$ 32.00 GB, System *Type*) 64-bit OS, $Operating\;System)$ Microsoft Windows 11 Pro. The settings of the proposed algorithms are presented in Table 1. For the HEA in phase 2, the population size is set to 500, and so is the maximum number of generations. To increase the chance of getting better approximations, the probability of mutation and crossover is set to 1, although that might negatively affect the computational time. All simulations are repeated 10 times and averaged to avoid any bias in the results. Despite its importance, the tuning task of the phase 2 HEA is not the core contribution of this work. The used parameters are set based on non-extensive simulations. As such, the obtained results are bounded by the performance of the experimental settings. Nonetheless, the tuning will likely affect the convergence speed and not the quality of the obtained approximations.

**Table 1:** Experimental settings.

Parameter	Value
*Initial population*	Concorde-Linkern
*Population size*	500
*Generations*	500
*Crossover*	PMX
*Mutation*	HC-MO
*Crossover probability*	1
*Mutation probability*	1
*Selection*	Tournament
*Encoding*	Permutation of integers
*HC-MO: iterations*	10
*HC-MO: neighborhood structure*	2-OPT
*PVNS:* $k_{min}$	1
*PVNS:* $k_{max}$	4
*PVNS: Neighborhood structures*	$\{1,2,3\}-$OPT, city insertion

### Test instances

We experiment 3PHEA on 20 bi-objective TSP instances available in the literature and summarized in Table 2. We consider four different types of instances:

- Euclidean instances: the costs between the edges correspond to the Euclidean distance between two points in a plane, randomly located from a uniform distribution (kroAB100, kroAC100, kroAD100, kroBC100, kroBD100, kroCD100, kroAB300, kroAB500, kroAB750, and kroAB1000).
- Random instances: the costs between the edges are randomly generated from a uniform distribution (randAB100, randCD100, and randEF100).
- Mixed instances: the first cost comes from the Euclidean instance while the second cost comes from the random instance (mixdGG100, mixdHH100, and mixdII100).
- Clustered instances: The points are randomly clustered in a plane, and the costs between the edges correspond to the Euclidean distance (clusAB100).

Lust’s datasets (L1–L10) have been used in Lust, and Teghem^60^ (instances L7–L10, also called the DIMACS instances) and in Lust and Teghem^41^ (instances L1–L6, also called the Krolak/Felts/Nelson instances—with prefix kro in TSPLIB). Paquete’s datasets have been used in Paquete, and Stützle^61^. Note that L7 is the same as P1, L9 is the same as P4, and finally, L10 is the same as P7. Lastly, Florios’ datasets (F1–F4) have been used in^48^. All datasets are available at^49^.

**Table 2:** The test bed of 16 datasets for the bi-objective TSP.

Lust’s instances	Name	Paquete’s instances	Name	Florios’ instances	Name
L1	kroAB100	P1	**euclAB100**	F1	kroAB300
L2	kroAC100	P2	euclCD100	F2	kroAB500
L3	kroAD100	P3	euclEF100	F3	kroAB750
L4	kroBC100	P4	**randAB100**	F4	kroAB1000
L5	kroBD100	P5	randCD100		
L6	kroCD100	P6	randEF100		
L7	**euclAB100**	P7	**mixdGG100**		
L8	clusAB100	P8	mixdHH100		
L9	**randAB100**	P9	mixdII100		
L10	**mixdGG100**				

Significant values are in [bold].

### Performance indicators

**Figure 5:**
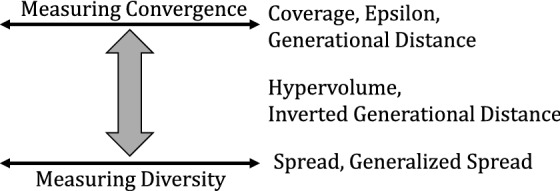
Taxonomy of multi-objective performance indicators.

Assessing the performance of metaheuristics in single-objective optimization is quite straightforward as it only requires comparing the best value obtained by an algorithm. For example, for a traditional TSP, the lower the length of a TSP circuit, the higher the algorithm is qualified. In contrast, in multi-objective optimization, the assessment becomes more complex as conflicting objectives are considered. For that, instead of a unique value, an approximation set to the true Pareto front is computed. In this convention, two properties are vital for assessment: (a) convergence toward the optimal front and (b) the diversity of the approximation set. To help measure the two properties, several quality metrics have been proposed in the literature. Among them are Coverage, Generational Distance, Inverse Generational Distance, Hypervolume, Epsilon, Spread, Generalized Spread, and others. Figure 5 depicts a classification of the assessment metrics. All the above-mentioned indicators have been considered in this work, and their implementation can be found here^62^. Since those indicators are subject to scaling issues, we apply them after normalizing the objective values. The used performance indicators can be defined as follows:

- **Coverage** ${\mathcal {C}}$: *C*(*X*, *Y*) represents the percentage of Pareto optimal solutions in set *Y* that are weakly dominated by a solution in set *X*.
  14 $$\begin{aligned} C(X, Y)= \frac{\left|\left\{ y \in Y \mid \exists \; x \in X: x \leqslant { }^{w} y\right\} \right|}{|Y |}, \end{aligned}$$
  the symbol $\leqslant { }^{w}$ represents weak dominance, that also holds true if $f(x)=f(y)$. A closer *C*(*AM*, *EPS*) to 1 indicates a better approximation set.
- **Generational distance** ${\mathcal {G}}$: ${\mathcal {G}}$ measures how far are the elements of an approximation set from those in the optimal Pareto front and is defined as:
  15 $$\begin{aligned} G(X, Y)=\frac{\sqrt{\sum _{i=1}^{|X |} d{(x_i,y_{x_i})}^{2}}}{|X |} \end{aligned}$$
  where $|X |$ is the size of the approximation set *X* and $d{(x_i,y_{x_i})}$ is the Euclidean distance measured in objective space between each solution $x \in X$ and its nearest solution $y_{x} \in Y$. A value of $G (X, Y) = 0$ indicates that all solutions in *X* are in the true Pareto front.
- **Inverse generational distance** $\mathcal{I}\mathcal{G}$: measures the distances between each solution in the Pareto front and its approximation. It can be defined as:
  16 $$\begin{aligned} IG(Y, X)=\frac{\sqrt{\sum _{i=1}^{|Y |} d{(y_i,x_{y_i})}^{2}}}{|Y |} \end{aligned}$$
  being $|Y |$ the number of solutions in the Pareto front and $d{(y_i,x_{y_i})}$ is the Euclidean distance between each solution in *Y* and its nearest neighbor in the approximation set *X*.
- **Hypervolume** ${\mathcal {H}}$: ${\mathcal {H}}$ computes the volume covered by a set of non-dominated solutions *X*.For each solution $x \in X$, a hypercube $v_x$ is constructed with respect to a reference point *W*. Subsequently, a union of all hypercubes is identified, and its hypervolume (*H*) is computed as follows:
  17 $$\begin{aligned} H={\text {volume}}\left( \bigcup _{i=1}^{|X |} v_{x_i}\right) \end{aligned}$$
- **Epsilon** ${\mathcal {E}}$: Given a computed front, *X*, ${\mathcal {E}}(X, Y)$ measures the smallest distance needed to translate every solution in *X* so that it dominates a solution in *Y*. More formally, given $\overrightarrow{x}=\left( x_{1}, \ldots , x_{n}\right)$ and $\overrightarrow{y}=\left( y_{1}, \ldots , y_{n}\right)$, where *n* is the number of objectives:
  18 $$\begin{aligned} {\mathcal {E}}_{\epsilon +}(X,Y)=\min _{\epsilon \in {\mathbb {R}}}\left\{ \forall \overrightarrow{y} \in Y ~ \exists \overrightarrow{x} \in X: \overrightarrow{x} \prec _{\epsilon } \overrightarrow{y}\right\} \end{aligned}$$
  where, $\overrightarrow{x} \prec _{\epsilon } \overrightarrow{y}$ if and only if $\forall ~ 1 \leqslant ~ i \leqslant n: x_{i}<y_{i}+\epsilon .$
- **Spread** ${\mathcal {S}}$: measures the spread of solutions in a given front. It is defined as:
  19 $$\begin{aligned} S(X)=\frac{(d_{r}+d_{l})+\sum _{i=1}^{|X|-1}\left|d_{i}-{\bar{d}}\right|}{(d_{r}+d_{l})+(|X|-1) * {\bar{d}}} \end{aligned}$$
  where $d_i$ is the Euclidean distance between consecutive solutions, ${\bar{d}}$ is the mean of distances, and $d_r$ and $d_l$ are the distances to the corner solutions. An *S* value equal to zero indicates an ideal distribution.
- **Generalized spread** $\mathcal{G}\mathcal{S}$: involves two sets, e.g., a true Pareto front and an approximation set. Formally, $\mathcal{G}\mathcal{S}$ can be computed as follows:
  20 $$\begin{aligned} GS(X,Y)=\frac{\sum _{i=1}^{m} d\left( e_{i}, X\right) +\sum _{i=1}^{|X|}|d_i-{\bar{d}}|}{\sum _{i=1}^{m} d\left( e_{i}, X\right) +|X|* {\bar{d}}} \end{aligned}$$
  where *X* is a set of solutions, *Y* is the set of Pareto optimal solutions, $(e_1,\ldots ,e_m)$ are *m* extreme solutions in *Y*, *m* is the number of objectives.

### Experimental analysis

**Table 3:** Experimental results for datasets (Lust and Teghem^41^).

Instance	Algorithm	$\mathcal {|PE |}$	$\mathcal {|D |}$	$\mathcal {|ND |\uparrow }$	${\mathcal {C}}\uparrow$	${\mathcal {H}}\uparrow$	${\mathcal {E}}\downarrow$	${\mathcal {G}}\downarrow$	$\mathcal{I}\mathcal{G}\downarrow$	${\mathcal {S}}\downarrow$	$\mathcal{G}\mathcal{S}\downarrow$	${\mathcal {T}}\downarrow$
*L1*	AUGM2	3332	0	3332	1	0.89944	0	0	0	0.78450	0.72851	134(h)
	2PPLS	2597	1043	1554	0.46638	0.89924	0.00157	7.428$e^{-6}$	8.056$e^{-6}$	0.80806	0.80712	25
	3PHEA$^1$	112	0	112	0.03361	0.89648	0.00905	1.237$e^{-6}$	1.332$e^{-4}$	0.64141	0.69672	23
	3PHEA$^2$	2585	1047	1538	0.46158	0.89922	0.00142	7.303$e^{-6}$	8.246$e^{-6}$	0.81072	0.80907	63
	3PHEA$^3$	3112	474	2638	0.79171	0.89939	0.00104	2.788$e^{-6}$	2.993$e^{-6}$	0.79156	0.75122	271
*L2*	AUGM2	2458	0	2458	1	0.89463	0	0	0	0.78522	0.75551	74(h)
	2PPLS	1971	748	1223	0.49755	0.89449	0.00125	6.541$e^{-6}$	7.641$e^{-6}$	0.75066	0.72904	22
	3PHEA$^1$	109	1	108	0.04393	0.89151	0.01020	3.528$e^{-6}$	1.706$e^{-4}$	0.60030	0.473824	22
	3PHEA$^2$	1975	662	1313	0.53417	0.89451	0.00109	6.082$e^{-6}$	7.395$e^{-6}$	0.74983	0.72990	48
	3PHEA$^3$	2278	326	1952	0.79414	0.89460	7.856$e^{-4}$	2.990$e^{-6}$	3.481$e^{-6}$	0.76232	0.73512	182
*L3*	AUGM2	2351	0	2351	1	0.87799	0	0	0	0.74683	0.62204	49(h)
	2PPLS	1808	820	988	0.42024	0.87778	0.00150	9.210$e^{-6}$	9.596$e^{-6}$	0.73723	0.65771	20
	3PHEA$^1$	86	2	84	0.03572	0.87312	0.01843	3.896$e^{-6}$	2.416$e^{-4}$	0.62121	0.63635	19
	3PHEA$^2$	1817	703	1114	0.47384	0.87782	0.00111	7.912$e^{-6}$	8.600$e^{-6}$	0.74192	0.68090	45
	3PHEA$^3$	2190	499	1691	0.71926	0.87793	9.070$e^{-4}$	4.815$e^{-6}$	4.377$e^{-6}$	0.73334	0.63818	180
*L4*	AUGM2	2752	0	2752	1	0.88609	0	0	0	0.72400	0.67578	77(h)
	2PPLS	2163	792	1371	0.49818	0.88597	0.00101	5.414$e^{-6}$	7.101$e^{-6}$	0.71736	0.64103	24
	3PHEA$^1$	118	1	117	0.04251	0.88348	0.00894	2.797$e^{-6}$	1.227$e^{-4}$	0.53310	0.55452	25
	3PHEA$^2$	2173	822	1351	0.49091	0.88597	8.663$e^{-4}$	5.503$e^{-6}$	7.145$e^{-6}$	0.70568	0.62213	52
	3PHEA$^3$	2598	363	2235	0.81213	0.88606	5.519$e^{-4}$	2.793$e^{-6}$	4.544$e^{-6}$	0.70910	0.65081	222

$\mathcal {|PE |}$: Number of potentially efficient solutions; $\mathcal {|D |}$: Number of dominated solutions; $\mathcal {|ND |}$: Number of nondominated solutions; ${\mathcal {C}}$: Coverage metric; ${\mathcal {H}}$: Hypervolume;  ${\mathcal {E}}$: Epsilon indicator;  ${\mathcal {G}}$: Generational distance;  $\mathcal{I}\mathcal{G}$: Inverted generational distance;  ${\mathcal {S}}$: Spread;  $\mathcal{G}\mathcal{S}$: Generalized Spread;  ${\mathcal {T}}$: Time in seconds unless specified.

**Table 4:** Experimental results for datasets (Lust and Teghem^41^).

Instance	Algorithm	$\mathcal {|PE |}$	$\mathcal {|D |}$	$\mathcal {|ND |\uparrow }$	${\mathcal {C}}\uparrow$	${\mathcal {H}}\uparrow$	${\mathcal {E}}\downarrow$	${\mathcal {G}}\downarrow$	$\mathcal{I}\mathcal{G}\downarrow$	${\mathcal {S}}\downarrow$	$\mathcal{G}\mathcal{S}\downarrow$	${\mathcal {T}}\downarrow$
*L5*	AUGM2	2657	0	2657	1	0.87819	0	0	0	0.75060	0.63876	66(h)
	2PPLS	2014	610	1404	0.52841	0.87806	0.00111	5.630$e^{-6}$	7.202$e^{-6}$	0.72806	0.62217	25
	3PHEA$^1$	116	2	114	0.04290	0.87511	0.01492	2.817$e^{-6}$	1.349$e^{-4}$	0.51931	0.52892	26
	3PHEA$^2$	1950	632	1318	0.49604	0.87804	0.00135	6.658$e^{-6}$	7.814$e^{-6}$	0.72007	0.61772	48
	3PHEA$^3$	2435	313	2122	0.79864	0.87817	6.347$e^{-4}$	2.280$e^{-6}$	2.660$e^{-6}$	0.73160	0.62488	202
*L6*	AUGM2	2044	0	2044	1	0.892617	0	0	0	0.71724	0.67429	39(h)
	2PPLS	1661	604	1057	0.51712	0.89245	0.00139	8.950$e^{-6}$	9.641$e^{-6}$	0.71049	0.66566	21
	3PHEA$^1$	100	0	100	0.04892	0.88916	0.01305	9.595$e^{-6}$	1.959$e^{-4}$	0.60198	0.60456	22
	3PHEA$^2$	1644	549	1095	0.53571	0.89247	0.00111	7.766$e^{-6}$	7.922$e^{-6}$	0.69311	0.65709	42
	3PHEA$^3$	1869	262	1607	0.78620	0.89258	7.580$e^{-4}$	3.477$e^{-6}$	3.845$e^{-6}$	0.70343	0.66572	156
*L7*	AUGM2	1812	0	1812	1	0.86268	0	0	0	0.65516	0.65440	41(h)
	2PPLS	1361	540	821	0.45309	0.86244	0.00180	1.196$e^{-5}$	1.244$e^{-5}$	0.60881	0.59882	20
	3PHEA$^1$	99	0	99	0.05463	0.85856	0.01420	5.167$e^{-6}$	2.176$e^{-4}$	0.56094	0.51750	21
	3PHEA$^2$	1352	493	859	0.47406	0.86247	0.00180	1.158$e^{-5}$	1.184$e^{-5}$	0.59432	0.58885	37
	3PHEA$^3$	1659	239	1420	0.78366	0.86263	8.625$e^{-4}$	4.228$e^{-6}$	4.629$e^{-6}$	0.62663	0.63310	141

$\mathcal {|PE |}$: Number of potentially efficient solutions;  $\mathcal {|D |}$: Number of dominated solutions;  $\mathcal {|ND |}$: Number of nondominated solutions;  ${\mathcal {C}}$: Coverage metric;  ${\mathcal {H}}$: Hypervolume;  ${\mathcal {E}}$: Epsilon indicator;  ${\mathcal {G}}$: Generational distance;  $\mathcal{I}\mathcal{G}$: Inverted generational distance;  ${\mathcal {S}}$: Spread;  $\mathcal{G}\mathcal{S}$: Generalized Spread;  ${\mathcal {T}}$: Time in seconds unless specified.

**Table 5:** Experimental results for datasets (Lust and Teghem^41^).

Instance	Algorithm	$\mathcal {|PE |}$	$\mathcal {|D |}$	$\mathcal {|ND |\uparrow }$	${\mathcal {C}}\uparrow$	${\mathcal {H}}\uparrow$	${\mathcal {E}}\downarrow$	${\mathcal {G}}\downarrow$	$\mathcal{I}\mathcal{G}\downarrow$	${\mathcal {S}}\downarrow$	$\mathcal{G}\mathcal{S}\downarrow$	${\mathcal {T}}\downarrow$
*L8*	AUGM2	3036	0	3036	1	0.91943	0	0	0	0.79422	0.73598	52(h)
	2PPLS	2535	996	1539	0.50691	0.91928	0.00151	6.943$e^{-6}$	7.549$e^{-6}$	0.80035	0.70292	30
	3PHEA$^1$	109	2	107	0.03524	0.91676	0.01010	6.565$e^{-6}$	1.481$e^{-4}$	0.634367	0.657556	34
	3PHEA$^2$	2450	969	1481	0.48781	0.91928	0.00179	6.931$e^{-6}$	7.996$e^{-6}$	0.81014	0.72327	60
	3PHEA$^3$	2854	439	2415	0.79545	0.91940	6.059$e^{-4}$	2.213$e^{-6}$	2.750$e^{-6}$	0.79915	0.73183	241
*L9*	AUGM2	1707	0	1707	1	0.92987	0	0	0	0.81921	0.76569	34(h)
	2PPLS	591	318	273	0.15992	0.92934	0.00200	2.780$e^{-5}$	4.890$e^{-5}$	0.76076	0.69889	19
	3PHEA$^1$	103	3	100	0.05858	0.92754	0.00960	9.738$e^{-6}$	2.272$e^{-4}$	0.70353	0.72012	21
	3PHEA$^2$	560	376	184	0.10779	0.92910	0.00273	3.705$e^{-5}$	6.084$e^{-5}$	0.77018	0.75326	39
	3PHEA$^3$	982	465	517	0.30287	0.92966	0.00142	1.377$e^{-5}$	2.782$e^{-5}$	0.81351	0.78045	85
*L10*	AUGM2	1848	0	1848	1	0.88571	0	0	0	0.67176	0.62277	38(h)
	2PPLS	970	409	561	0.30357	0.88531	0.00213	1.669$e^{-5}$	2.522$e^{-5}$	0.66163	0.66229	24
	3PHEA$^1$	124	7	117	0.06331	0.88251	0.01171	3.670$e^{-5}$	1.632$e^{-4}$	0.55044	0.59859	25
	3PHEA$^2$	954	386	568	0.30735	0.88522	0.00213	1.680$e^{-5}$	3.512$e^{-5}$	0.71755	0.71447	35
	3PHEA$^3$	1378	332	1046	0.56601	0.88559	0.00152	7.057$e^{-6}$	1.316$e^{-5}$	0.67008	0.62539	110

$\mathcal {|PE |}$: Number of potentially efficient solutions;  $\mathcal {|D |}$: Number of dominated solutions;  $\mathcal {|ND |}$: Number of nondominated solutions;  ${\mathcal {C}}$: Coverage metric;  ${\mathcal {H}}$: Hypervolume;  ${\mathcal {E}}$: Epsilon indicator;  ${\mathcal {G}}$: Generational distance;  $\mathcal{I}\mathcal{G}$: Inverted generational distance;  ${\mathcal {S}}$: Spread;  $\mathcal{G}\mathcal{S}$: Generalized Spread;  ${\mathcal {T}}$: Time in seconds unless specified.

Tables 3, 4, and 5 present the experimental results of the proposed 3PHEA method compared to two other approaches from the literature AUGMECON2 (AUGM2)^48^ and 2PPLS^41^ for ten multi-objective TSP instances (L1–L10). AUGM2 is an exact approach for multi-objective problems based on an improved epsilon constraint and branch and cut techniques; it efficiently computes the true Pareto front for a given instance of a Multi-Objective Integer Programming problem such as TSP or Set Covering. On the other hand, 2PPLS is an improved heuristic based on a Pareto local search procedure dedicated to multi-objective TSP and results in an approximated Pareto front. For 3PHEA, we report the cumulative results of the three phases to assess the contribution of each phase. For example, 3PHEA$^2$ indicates the results of applying phase 1 followed by phase 2; likewise, 3PHEA$^3$ comprises the three phases and thus is the final outcome of our method.

**Figure 6:**
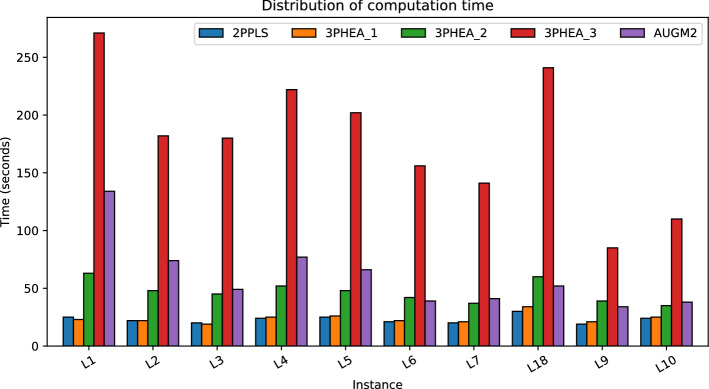
Distribution of computation time of different algorithms for datasets (Lust and Teghem^41^).

As can be seen from Tables 3, 4, and 5, the computational time of AUGM2 is significantly high, ranging from 34 to 134 h depending on the instance. Interestingly, AUGM2 execution time is often exponentially proportional to the size of the Pareto front $\mathcal {|PE |}$. For L1, with 3332 non-dominated solutions, the execution time is 134 h, while it decreases to 32 h for L9 having $\mathcal {|PE |}=1707$. With that, AUGM2 might drastically suffer from high computation time for instances with many non-dominated solutions, even though the instance size is small. As for 2PPLS, the computation time is relatively low, ranging between 20 and 30 s, and the coverage metric varies between 0.15 and 0.52. For example, for L9, which is a randomly generated instance, 2PPLS only found 273 among the 1707 Pareto solutions; the coverage improved but was still relatively low for L10, which has a mixture of random and euclidean objective values; lastly, for the euclidean instances, 2PPLS coverage relatively improves to reach 0.52 such as in instance L5. Those results obviously indicate that the local search procedure in 2PPLS might rapidly fall into local minima due to the random nature of the instance. As far as 3PHEA is concerned, the computation time follows a similar trend compared to AUGM2 and 2PPLS. That is, the computation time is closely linked with the size of the true Pareto front. By assessing the contribution of each phase, we can deduce that among the three phases of 3PHEA, the third phase related to the Pareto VNS takes most of the computation time. That is mainly due to the number of neighborhood functions and their corresponding neighborhood size. The distribution of computation time of all methods is reported in Fig. 6.

**Figure 7:**
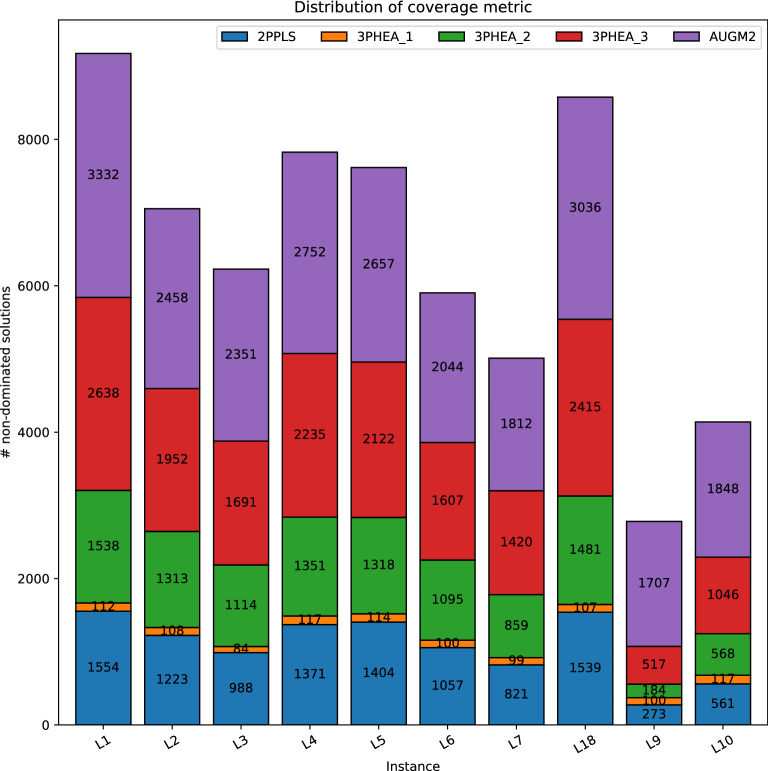
Distribution of coverage metric for datasets (Lust and Teghem^41^).

Assessing the coverage metric indicates that 3PHEA could significantly approximate the true Pareto front compared to 2PPLS. Specifically, 3PHEA achieves up to 81% of the non-dominated solutions compared to 49% resulting from 2PPLS (see instance L4 in Table 3). To assess the contribution of each of the three phases’ contribution, we plot the coverage metric distribution in Fig. 7. As can be seen, the contribution of phase 3 PVNS is the largest compared to phase 1 and phase 2. Nonetheless, the results of PVNS substantially depend on phases 1 and 2. The superiority of 3PHEA is also reflected by the rest of the multi-objective indicators as indicated in Tables 3, 4, and 5. For example, regarding the ${\mathcal {H}}$ hypervolume indicator, results show that the value obtained by our method is always higher than the value obtained by 2PPLS, indicating that our obtained fronts are much closer to the true Pareto fronts and better cover solutions residing in the extremities. Moreover, the values of the indicators ${\mathcal {E}}$, ${\mathcal {G}}$, $\mathcal{I}\mathcal{G}$, ${\mathcal {S}}$, and $\mathcal{G}\mathcal{S}$ of our method are usually lower than 2PPLS indicating a closer results to the true Pareto front, and better distribution of the obtain non-dominated solutions. With the latter result, it can be concluded tat our method has a notable search feature that does not only guide the algorithm to the true Pareto front, but it also balances, thanks to the exploitation and exploration properties, the search so that it covers the entire regions in the solution space. That is crucial to allow the decision makers to select the most suitable non-dominated points.

Experimental results for the datasets of (Stützle and Paquete^61^) are presented in Tables 6 and 7. As can be seen, for P2 and P3 instances, the coverage metric of the 3PHEA approach rises to 76% and 78%, respectively. 3PHEA could also considerably result in a better approximation for random and mixed instances (P5 and P6) and mixed instances (P8 and P9). The multi-objective indicators reflect the dominant performance of 3PHEA compared to 2PPLS. Nevertheless, the improvement in quality increases the computation time of 3PHEA. It is worth noting that the coverage of 3PHEA for those instances is also proportionally linked to the size of the true Pareto front. For example, for P3, $\mathcal {|ND^*|}=2530$, the coverage of 3PHEA is 78%, while it decreases to 24% for P5 having $\mathcal {|ND^* |}=1850$.

The results of larger instances F1, F2, F3, and F4 (Florios and Mavrotas^48^) corresponding to 300, 500, 750, and 1000 cities, respectively, are presented in Table 8. Given a large number of neighborhood solutions, we only consider two neighbor structures, 1OPT, and 2OPT, for instances F3 and F4; otherwise, the computation time will be terribly significant. It is worth noting that for all the instances in this table, the AUGM2 method, which is still the reference method for 2PPLS and 3PHEA, does not provide the true Pareto front due to its high computation time. Therefore, the true Pareto fronts of these instances are not yet known in the literature. Instead, AUGM2 simply uses an approximation approach based on Pareto local search and simulated annealing and repeats its execution 20 times to generate an elite population of approximate efficient solutions; the computation time of AUGM2 is not reported in its original paper^48^. Results indicate that 3PHEA has a coverage value $>1$, meaning that 3PHEA could find new non-dominated solutions that have not been found by AUGM2. For example, for instance F1, 3PHEA finds 16,209 non-dominated solutions while AUGM2 finds 14,867 and 2PPLS finds 10,295. It can also be noticed that for F3 and F4 instances, the coverage of 3PHEA is slightly less than AUGM2 due to the absence of 3OPT and city insertion neighbor structures. Nonetheless, 3PHEA considerably outperforms 2PPLS regarding the various multi-objective indicators. Regarding the computation complexity, results point out that large instances have Pareto fronts of larger size and thereby drastically affect the execution time of all methods. For example, for F4 with 1000 cities, the execution time of 3PHEA might increase to 12,604 s. The time will be even more critical when more neighbor structures are considered.

**Table 6:** Experimental results for datasets (Stützle and Paquete^61^).

Instance	Algorithm	$\mathcal {|PE |}$	$\mathcal {|D |}$	$\mathcal {|ND |\uparrow }$	${\mathcal {C}}\uparrow$	${\mathcal {H}}\uparrow$	${\mathcal {E}}\downarrow$	${\mathcal {G}}\downarrow$	$\mathcal{I}\mathcal{G}\downarrow$	${\mathcal {S}}\downarrow$	$\mathcal{G}\mathcal{S}\downarrow$	${\mathcal {T}}\downarrow$
*P2*	AUGM2	2268	0	2268	1	0.85920	0	0	0	0.64159	0.62519	67(h)
	2PPLS	1645	628	1017	0.4484	0.85899	0.00183	8.974$e^{-6}$	9.318$e^{-6}$	0.60053	0.58656	21
	3PHEA$^1$	101	2	99	0.04365	0.85489	0.01393	8.748$e^{-6}$	1.884$e^{-4}$	0.57171	0.616403	20
	3PHEA$^2$	0 1634	627	1007	0.44400	0.85895	0.00183	1.030$e^{-5}$	1.015$e^{-5}$	0.59218	0.57230	42
	3PHEA$^3$	2064	334	1730	0.76278	0.85914	7.483$e^{-4}$	3.725$e^{-6}$	4.473$e^{-6}$	0.63134	0.61131	173
*P3*	AUGM2	2530	0	2530	1	0.86372	0	0	0	0.76401	0.80429	52(h)
	2PPLS	1932	707	1225	0.48418	0.86352	0.00127	7.629$e^{-6}$	9.195$e^{-6}$	0.77127	0.84370	23
	3PHEA$^1$	107	3	104	0.04110	0.85953	0.01795	1.066$e^{-5}$	1.763$e^{-4}$	0.58356	0.53867	22
	3PHEA$^2$	2003	745	1258	0.49723	0.86342	0.00227	1.072$e^{-5}$	1.077$e^{-5}$	0.75384	0.82208	50
	3PHEA$^3$	2330	348	1982	0.78339	0.86364	0.00113	4.018$e^{-6}$	4.630$e^{-6}$	0.76314	0.81620	194
*P5*	AUGM2	1850	0	1850	1	0.92464	0	0	0	0.78841	0.75808	39(h)
	2PPLS	584	312	272	0.14702	0.92405	0.00255	3.216$e^{-5}$	4.534$e^{-5}$	0.71296	0.67090	23
	3PHEA$^1$	100	1	99	0.05351	0.92251	0.00650	1.608$e^{-6}$	1.980$e^{-4}$	0.62090	0.65822	22
	3PHEA$^2$	558	383	175	0.09459	0.92384	0.00255	3.985$e^{-5}$	6.727$e^{-5}$	0.74978	0.67900	38
	3PHEA$^3$	1002	546	456	0.24648	0.92435	0.00156	1.564$e^{-5}$	2.808$e^{-5}$	0.78663	0.76163	84

$\mathcal {|PE |}$: Number of potentially efficient solutions;  $\mathcal {|D |}$: Number of dominated solutions;  $\mathcal {|ND |}$: Number of nondominated solutions;  ${\mathcal {C}}$: Coverage metric;  ${\mathcal {H}}$: Hypervolume;  ${\mathcal {E}}$: Epsilon indicator;  ${\mathcal {G}}$: Generational distance;  $\mathcal{I}\mathcal{G}$: Inverted generational distance;  ${\mathcal {S}}$: Spread;  $\mathcal{G}\mathcal{S}$: Generalized Spread;  ${\mathcal {T}}$: Time in seconds unless specified.

**Table 7:** Experimental results for datasets (Stützle and Paquete^61^).

Instance	Algorithm	$\mathcal {|PE |}$	$\mathcal {|D |}$	$\mathcal {|ND |\uparrow }$	${\mathcal {C}}\uparrow$	${\mathcal {H}}\uparrow$	${\mathcal {E}}\downarrow$	${\mathcal {G}}\downarrow$	$\mathcal{I}\mathcal{G}\downarrow$	${\mathcal {S}}\downarrow$	$\mathcal{G}\mathcal{S}\downarrow$	${\mathcal {T}}\downarrow$
*P6*	AUGM2	1882	0	1882	1	0.92691	0	0	0	0.79438	0.75538	44(h)
	2PPLS	672	337	335	0.17800	0.92648	0.00191	2.434$e^{-5}$	3.903$e^{-5}$	0.738221	0.66037	24
	3PHEA$^1$	134	6	128	0.06801	0.92496	0.00903	2.554$e^{-5}$	1.845$e^{-4}$	0.74629	0.70672	27
	3PHEA$^2$	617	398	219	0.11636	0.92624	0.00226	3.141$e^{-5}$	4.232$e^{-5}$	0.78937	0.75881	39
	3PHEA$^3$	1048	475	573	0.30446	0.92672	0.00106	1.127$e^{-5}$	2.109$e^{-5}$	0.78120	0.71424	83
*P8*	AUGM2	2108	0	2108	1	0.88897	0	0	0	0.72914	0.71297	35(h)
	2PPLS	1171	509	662	0.31404	0.88866	0.00220	1.294$e^{-5}$	2.069$e^{-5}$	0.75537	0.76489	26
	3PHEA$^1$	126	4	122	0.05787	0.88632	0.00854	1.322$e^{-5}$	1.502$e^{-4}$	0.55913	0.56540	27
	3PHEA$^2$	1130	521	609	0.28889	0.88855	0.00180	1.464$e^{-5}$	2.534$e^{-5}$	0.75824	0.75657	37
	3PHEA$^3$	1609	362	1247	0.59155	0.88886	8.818$e^{-4}$	5.315$e^{-6}$	1.009$e^{-5}$	0.73055	0.73913	135
*P9*	AUGM2	1883	0	1883	1	0.89846	0	0	0	0.75624	0.71879	40(h)
	2PPLS	959	439	520	0.27615	0.89921	9.593$e^{-4}$	3.102$e^{-5}$	4.208$e^{-5}$	0.75868	0.75449	22
	3PHEA$^1$	108	0	108	0.05735	0.89670	0.01066	1.390$e^{-4}$	1.956$e^{-4}$	0.63043	0.62255	23
	3PHEA$^2$	995	507	488	0.25916	0.89910	0.00100	2.799$e^{-5}$	4.558$e^{-5}$	0.76261	0.75635	34
	3PHEA$^3$	1452	384	1068	0.56718	0.89948	2.099$e^{-4}$	2.648$e^{-5}$	2.648$e^{-5}$	0.77585	0.74987	125

$\mathcal {|PE |}$: Number of potentially efficient solutions;  $\mathcal {|D |}$: Number of dominated solutions;  $\mathcal {|ND |}$: Number of nondominated solutions;  ${\mathcal {C}}$: Coverage metric;  ${\mathcal {H}}$: Hypervolume;  ${\mathcal {E}}$: Epsilon indicator;  ${\mathcal {G}}$: Generational distance;  $\mathcal{I}\mathcal{G}$: Inverted generational distance;  ${\mathcal {S}}$: Spread;  $\mathcal{G}\mathcal{S}$: Generalized Spread;  ${\mathcal {T}}$: Time in seconds unless specified.

**Table 8:** Experimental results for datasets (Florios and Mavrotas^48^).

Instance	Algorithm	$\mathcal {|PE |}$	$\mathcal {|D |}$	$\mathcal {|ND |\uparrow }$	${\mathcal {C}}\uparrow$	${\mathcal {H}}\uparrow$	${\mathcal {E}}\downarrow$	${\mathcal {G}}\downarrow$	$\mathcal{I}\mathcal{G}\downarrow$	${\mathcal {S}}\downarrow$	$\mathcal{G}\mathcal{S}\downarrow$	${\mathcal {T}}\downarrow$
*F1*	AUGM2	14867	0	14867	1	0.91922	0	0	0	0.73907	0.73374	–
	2PPLS	15092	4797	10295	0.69247	0.91863	9.361$e^{-4}$	4.659$e^{-6}$	4.548$e^{-6}$	0.74458	0.75550	205
	3PHEA	16637	428	16209	1.09026	0.91868	7.080$e^{-4}$	4.104$e^{-6}$	4.194$e^{-6}$	0.75073	0.73503	1497
*F2*	AUGM2	33929	0	33929	1	0.92829	0	0	0	0.73144	0.67604	–
	2PPLS	1932	707	1225	0.48418	0.86352	0.00127	7.629$e^{-6}$	9.195$e^{-6}$	0.77127	0.84370	458
	3PHEA	38109	3143	34966	1.03056	0.92838	8.460$e^{-5}$	4.987$e^{-7}$	5.308$e^{-7}$	0.73248	0.68708	7164
*F3*	AUGM2	61184	0	61184	1	0.93790	0	0	0	0.75048	0.69552	–
	2PPLS	60657	20831	39826	0.65092	0.92986	6.345$e^{-5}$	7.548$e^{-6}$	7.865$e^{-6}$	0.75938	0.70144	3256
	3PHEA	67301	9488	57813	0.94490	0.93687	4.411$e^{-5}$	7.289$e^{-6}$	7.752$e^{-6}$	0.77034	0.71571	8803
*F4*	AUGM2	98151	0	98151	1	0.94433	0	0	0	0.78683	0.70874	–
	2PPLS	45120	0	45120	0.45969	0.91174	6.345$e^{-5}$	7.548$e^{-6}$	7.865$e^{-6}$	0.75938	0.70144	7462
	3PHEA	95128	7872	87256	0.88899	0.93543	4.411$e^{-5}$	7.289$e^{-6}$	7.752$e^{-6}$	0.77034	0.71571	12604

$\mathcal {|PE |}$: Number of potentially efficient solutions;  $\mathcal {|D |}$: Number of dominated solutions;  $\mathcal {|ND |}$: Number of nondominated solutions;  ${\mathcal {C}}$: Coverage metric;  ${\mathcal {H}}$: Hypervolume;  ${\mathcal {E}}$: Epsilon indicator;  ${\mathcal {G}}$: Generational distance;  $\mathcal{I}\mathcal{G}$: Inverted generational distance;  ${\mathcal {S}}$: Spread;  $\mathcal{G}\mathcal{S}$: Generalized Spread;  ${\mathcal {T}}$: Time in seconds unless specified.

To statistically analyze the studied methods, we compare in the following the variations in the results of our proposed algorithm 3PHEA against the 2PPLS method. We use the non-parametric Mann-Whitney U test (also called the Wilcoxon–Mann–Whitney test)^63^. In this test, we aim to compare the distributions of the multi-objective performance indicators of 3PHEA and 2PPLS. The null hypothesis is stated as follows: “$H_0$: the two samples derive from identical populations“ for the performance indicators ${\mathcal {H}}$, ${\mathcal {E}}$, ${\mathcal {G}}$, $\mathcal{I}\mathcal{G}$, ${\mathcal {S}}$, $\mathcal{G}\mathcal{S}$ on a given instance. We use the equality symbol $=$ if *H*0 is satisfied. In this case, we conclude that the two algorithms perform similarly regarding a performance indicator. Contrarily, when $H_0$ is not satisfied, we conclude that the two algorithms perform differently concerning a performance indicator. In this case, we rely on the mean value to compare the two algorithms. That is, we use the > sign if the mean value obtained via our algorithm 3PHEA is better than the one obtained via 2PPLS. Similarly, we use the < sign if the mean value of 3PHEA is worse than that of 2PPLS. The hypothesis of each of the seven performance metrics is tested concurrently with an alpha level equal to .05.

The statistical results are reported in Table 9. As can be seen from Table 9, the statistical test indicates with a low risk that our method 3PHEA outperforms 2PPLS in all test instances regarding ${\mathcal {C}}$, ${\mathcal {H}}$, ${\mathcal {E}}$, ${\mathcal {G}}$, $\mathcal{I}\mathcal{G}$. This proves the superior quality of the 3PHEA Pareto fronts in terms of their closeness to the True Pareto fronts of the various instances. Moreover, it can be argued that the results of 3PHEA are stable, making the approach robust regardless of the instance, the structure of the initial population, or the impact of evolutionary operators involved in the three phases. Increasing the size of instances does not affect the statistical performance of 3PHEA regarding the above-mentioned indicators. That emphasizes the ability of 3PHEA to handle different solutions landscapes. Differently, statistical results for ${\mathcal {S}}$ and $\mathcal{G}\mathcal{S}$ do not follow a consistent pattern. Table 9 indicates that 2PPLS is superior to 3PHEA for some test instances e.g., L2, L5, L7, P2, etc. which means that the distribution of solutions in the obtained Pareto fronts of 2PPLS might sometimes be of better quality. That is, solutions are well-distributed and are not concentrated on a specific region in the front. However, that conclusion, when analyzed alone, is of lower importance, given that a well-distributed front is only relevant if it is close to the true Pareto front. In other words, for an algorithm to be superior, its results must reflect solid exploitation and exploration features, such that the most promising regions in the solution space of a given problem are adequately represented.

**Table 9:** Wilcoxon–Mann–Whitney statistical test for 3PHEA against 2PPLS.

Instance	${\mathcal {C}}$	${\mathcal {H}}$	${\mathcal {E}}$	${\mathcal {G}}$	$\mathcal{I}\mathcal{G}$	${\mathcal {S}}$	$\mathcal{G}\mathcal{S}$
L1	>	>	>	>	>	>	>
L2	>	>	>	>	>	<	<
L3	>	>	>	>	>	$=$	>
L4	>	>	>	>	>	>	<
L5	>	>	>	>	>	<	$=$
L6	>	>	>	>	>	>	$=$
L7	>	>	>	>	>	<	<
L8	>	>	>	>	>	>	<
L9	>	>	>	>	>	<	<
L10	>	>	>	>	>	<	>
P2	>	>	>	>	>	<	<
P3	>	>	>	>	>	>	>
P5	>	>	>	>	>	<	<
P6	>	>	>	>	>	<	<
P8	>	$=$	>	>	>	>	>
P9	>	>	>	>	>	<	>
F1	>	>	>	>	>	<	>
F2	>	>	>	>	>	>	>
F3	>	>	>	>	>	<	<
F4	>	>	>	>	>	<	<

## Discussions

Experimental results indicate that the performance of the proposed 3PHEA is relatively acceptable for solving bi-objective TSP compared to an exact and approximation method. To help with the decision-making process, the trade-off solutions can be grouped into *k* clusters covering the totality of the Pareto front and thereafter ranked based on their robustness. The latter is crucial as the travel time might vary depending on the traffic situation, weather conditions, traveler’s profile, etc. Integrating real-time traffic settings thus helps decision-makers select a subset of robust solutions from many non-comparable points. Results also indicate that the third phase of 3PHEA might be time-consuming, mainly due to the usage of multiple neighborhood structures. This issue will be even more prominent when the size of the problem becomes essential or the number of objectives increases. To tackle this problem, we discuss the following. Neighborhood structures should be distinct but complementary in the sense that the same solution if found via multiple structures, must only be processed one time; if not, additional time overhead will be added to the process and thereby drastically impact the computational time. Furthermore, visual analysis of trade-off solutions indicates that some edges are repeated across different solutions. Those edges might be identified a priori as “elite edges” using a machine learning model and dynamically utilized during the local search procedures. The knowledge about the decision and objective spaces can be analyzed and taken even further by predicting whether applying a local search (HC-MO to PVNS) on a given input using a neighborhood structure is promising or not (i.e., leading to new dominant or non-dominated solutions); we have recently made solid progress in that direction (see^64^); speed up techniques could also be considered here (see^65^).

Even though we did not conduct extensive parameters tuning analysis, the proposed algorithms performed relatively well. Deciding on the optimal input parameters, such as the population size in phase 2, the neighborhood structures, and their order in phase 3, remains an open question. Experimental results in that direction are one of our future works. In addition, we plan to extend our approach to handle many objectives. For that, phase 1 should be augmented to cope with many-dimensional shapes; for phases 2 and 3, we believe their adaptation is relatively straightforward (Supplementary Information).

## Conclusions

This paper studied the Bi-objective Traveling Salesman Problem (BTSP), where two conflicting objectives, travel time and monetary cost, were considered under minimization. To efficiently solve the BTSP, we introduced a novel three-Phase Hybrid Evolutionary Algorithm based on the Lin–Kernighan Heuristic, an improved version of the Non-Dominated Sorting Genetic Algorithm and Pareto Variable Neighborhood Search. We assessed the performance of the proposed approach by solving 20 real-world TSP instances of various degrees of difficulty and sizes ranging from 100 to 1000 cities. We also computed several multi-objective performance indicators, including running time, coverage, hypervolume, epsilon, generational distance, inverted generational distance, spread, and generalized spread. We compared our method with three existing algorithms from the literature, an exact approach based on epsilon constraint and branch and cut, and two approximation approaches based on Pareto local search and simulated annealing. Experimental results indicate that our method is significantly superior to existing approaches covering up to 80% of the actual Pareto fronts. For future works, we plan to incorporate more criteria in our optimization model, such as the capacity of vehicles and uncertain travel time. In addition, we aim to optimize the time complexity of the neighborhood structures via a machine-learning module and compare our approach with more multi-objective evolutionary algorithms.

## Supplementary Information

Supplementary Information.

## Data Availability

All data generated or analyzed during this study are included in this published article [and its supplementary information files].

## References

[1] Matai R, Singh SP, Mittal ML (2010). Traveling salesman problem: An overview of applications, formulations, and solution approaches. Travel. Salesman Probl. Theory Appl..

[2] Alexandridis A, Paizis E, Chondrodima E, Stogiannos M (2017). A particle swarm optimization approach in printed circuit board thermal design. Integrat. Comput. Aided Eng..

[3] Nalecz-Charkiewicz K, Nowak RM (2022). Algorithm for dna sequence assembly by quantum annealing. BMC Bioinform..

[4] Carpio, R. F., Maiolini, J., Potena, C., Garone, E., Ulivi, G., Gasparn, A. Mp-stsp: A multi-platform steiner traveling salesman problem formulation for precision agriculture in orchards. In *2021 IEEE International Conference on Robotics and Automation (ICRA)*, pp 2345–2351 (2021). 10.1109/ICRA48506.2021.9561023.

[5] Mosayebi M, Sodhi M, Wettergren TA (2021). The traveling salesman problem with job-times (tspj). Comput. Oper. Res..

[6] Dodge M, MirHassani S, Hooshmand F (2021). Solving two-dimensional cutting stock problem via a dna computing algorithm. Nat. Comput..

[7] Ilavarasi, K., Joseph, K. S. Variants of travelling salesman problem: A survey. In *International Conference on Information Communication and Embedded Systems (ICICES2014)*, pp. 1–7 (2014). 10.1109/ICICES.2014.7033850.

[8] Khan I, Maiti MK, Basuli K (2020). Multi-objective traveling salesman problem: An abc approach. Appl. Intell..

[9] Defryn C, Sörensen K (2018). Multi-objective optimisation models for the travelling salesman problem with horizontal cooperation. Eur. J. Oper. Res..

[10] Deb K (2014). Multi-objective optimization. Search Methodol..

[11] Liu Q, Li X, Liu H, Guo Z (2020). Multi-objective metaheuristics for discrete optimization problems: A review of the state-of-the-art. Appl. Soft Comput..

[12] Liu S-C, Zhan Z-H, Tan KC, Zhang J (2021). A multiobjective framework for many-objective optimization. IEEE Trans. Cybern..

[13] Dahiya C, Sangwan S (2018). Literature review on travelling salesman problem. Int. J. Res..

[14] Osaba E, Yang X-S, Del Ser J (2020). Traveling salesman problem: A perspective review of recent research and new results with bio-inspired metaheuristics. Nat. Inspired Comput. Swarm Intell..

[15] Hussain K, Salleh MNM, Cheng S, Shi Y (2019). Metaheuristic research: A comprehensive survey. Artif. Intell. Rev..

[16] Kowalski M, Izdebski M, Żak J, Gołda P, Manerowski J (2021). Planning and management of aircraft maintenance using a genetic algorithm. Eksploatacja Niezawodność.

[17] Amal L, Son LH, Chabchoub H (2018). Sga: Spatial gis-based genetic algorithm for route optimization of municipal solid waste collection. Environ. Sci. Pollut. Res..

[18] Akpunar ÖŞ, Akpinar Ş (2021). A hybrid adaptive large neighbourhood search algorithm for the capacitated location routing problem. Expert Syst. Appl..

[19] Sadati MEH, Çatay B, Aksen D (2021). An efficient variable neighborhood search with tabu shaking for a class of multi-depot vehicle routing problems. Comput. Oper. Res..

[20] Pourghasemi HR, Razavi-Termeh SV, Kariminejad N, Hong H, Chen W (2020). An assessment of metaheuristic approaches for flood assessment. J. Hydrol..

[21] Dib O, Manier M-A, Moalic L, Caminada A (2017). Combining vns with genetic algorithm to solve the one-to-one routing issue in road networks. Comput. Oper. Res..

[22] Dib O, Moalic L, Manier M-A, Caminada A (2017). An advanced ga-vns combination for multicriteria route planning in public transit networks. Expert Syst. Appl..

[23] Dib O, Dib M, Caminada A (2018). Computing multicriteria shortest paths in stochastic multimodal networks using a memetic algorithm. Int. J. Artif. Intell. Tools.

[24] Zhao, P., Xu, D. Hybrid algorithm for solving traveling salesman problem. In *IOP Conference Series: Materials Science and Engineering*, vol 646, p. 012032 (2019). 10.1088/1757-899X/646/1/012032.

[25] Gunantara N (2018). A review of multi-objective optimization: Methods and its applications. Cogent Eng..

[26] Deb K, Pratap A, Agarwal S, Meyarivan T (2002). A fast and elitist multiobjective genetic algorithm: Nsga-ii. IEEE Trans. Evol. Comput..

[27] Wang S, Zhao D, Yuan J, Li H, Gao Y (2019). Application of nsga-ii algorithm for fault diagnosis in power system. Electr. Power Syst. Res..

[28] Sun Y, Lin F, Xu H (2018). Multi-objective optimization of resource scheduling in fog computing using an improved nsga-ii. Wirel. Pers. Commun..

[29] Saikia R, Sharma D (2021). Reference-lines-steered memetic multi-objective evolutionary algorithm with adaptive termination criterion. Memetic Comput..

[30] Garcia-Garcia, C., Martínez-Peñaloza, M.-G., Morales-Reyes, A. cmoga/d: A novel cellular ga based on decomposition to tackle constrained multiobjetive problems. In: *Proceedings of the 2020 Genetic and Evolutionary Computation Conference Companion*, pp. 1721–1729 (2020). 10.1145/3377929.3398137.

[31] Hu W, Fathi M, Pardalos PM (2018). A multi-objective evolutionary algorithm based on decomposition and constraint programming for the multi-objective team orienteering problem with time windows. Appl. Soft Comput..

[32] Liang Z, Luo T, Hu K, Ma X, Zhu Z (2020). An indicator-based many-objective evolutionary algorithm with boundary protection. IEEE Trans. Cybern..

[33] Bai WS, Guo XW, Peng F, Qi L, jin Qin S (2021). An s-metric selection evolutionary multi-objective optimization algorithm solving u-shaped disassembly line balancing problem. J. Phys. Conf. Ser..

[34] Luo J, Yang Y, Li X, Liu Q, Chen M, Gao K (2018). A decomposition-based multi-objective evolutionary algorithm with quality indicator. Swarm Evol. Comput..

[35] Li F, Cheng R, Liu J, Jin Y (2018). A two-stage r2 indicator based evolutionary algorithm for many-objective optimization. Appl. Soft Comput..

[36] Bechikh S, Chaabani A, Ben Said L (2015). An efficient chemical reaction optimization algorithm for multiobjective optimization. IEEE Trans. Cybern..

[37] Deb, K., Thiele, L., Laumanns, M., Zitzler, E. Scalable test problems for evolutionary multiobjective optimization. In *Evolutionary Multiobjective Optimization*, pp. 105–145 (2005). 10.1007/1-84628-137-7_6.

[38] Dhiman G, Singh KK, Slowik A, Chang V, Yildiz AR, Kaur A, Garg M (2021). Emosoa: A new evolutionary multi-objective seagull optimization algorithm for global optimization. Int. J. Mach. Learn. Cybern..

[39] Siddiqi, F. A., & Mofizur Rahman, C. Evolutionary multi-objective whale optimization algorithm, pp. 431–446 (2020). 10.1007/978-3-030-16660-1_43.

[40] Zitzler E, Deb K, Thiele L (2000). Comparison of multiobjective evolutionary algorithms: Empirical results. Evol. Comput..

[41] Lust T, Teghem J (2010). Two-phase pareto local search for the biobjective traveling salesman problem. J. Heuristics.

[42] Applegate, D., Bixby, R., Chvatal, V., Cook, W. Concorde TSP solver (2006). http://www.tsp.gatech.edu/concorde.

[43] Paquete, L., Chiarandini, M., Stützle, T. Pareto local optimum sets in the biobjective traveling salesman problem: An experimental study. In *Metaheuristics for Multiobjective Optimisation*, pp. 177–199 (2004). 10.1007/978-3-642-17144-4_7.

[44] Lust, T. Multiobjective TSP. https://sites.google.com/site/thibautlust/research/multiobjective-tsp (2009).

[45] de Carvalho EB, Goldbarg EFG, Goldbarg MC (2018). A multi-objective version of the lin-kernighan heuristic for the traveling salesman problem. Rev. Inform. Teórica Apl..

[46] Costa L, Lust T, Kramer R, Subramanian A (2018). A two-phase pareto local search heuristic for the bi-objective pollution-routing problem. Networks.

[47] Zhou Q, Wang J, Zhang G, Guo K, Cai X, Wang L, Huang Y (2019). A two-phase multiobjective local search for the device allocation in the distributed integrated modular avionics. IEEE Access.

[48] Florios K, Mavrotas G (2014). Generation of the exact pareto set in multi-objective traveling salesman and set covering problems. Appl. Math. Comput..

[49] Florios, K. Multiobjective traveling salesman problem (MOTSP). https://sites.google.com/site/kflorios/motsp (2021).

[50] Mahrach M, Miranda G, León C, Segredo E (2020). Comparison between single and multi-objective evolutionary algorithms to solve the knapsack problem and the travelling salesman problem. Mathematics.

[51] Moraes DH, Sanches DS, da Silva Rocha J, Garbelini JMC, Castoldi MF (2019). A novel multi-objective evolutionary algorithm based on subpopulations for the bi-objective traveling salesman problem. Soft Comput..

[52] Agrawal A, Ghune N, Prakash S, Ramteke M (2021). Evolutionary algorithm hybridized with local search and intelligent seeding for solving multi-objective euclidian tsp. Expert Syst. Appl..

[53] Michalak K (2021). Evolutionary algorithm using random immigrants for the multiobjective travelling salesman problem. Proced. Comput. Sci..

[54] Tinós, R., Helsgaun, K., Whitley, D. Efficient recombination in the lin-kernighan-helsgaun traveling salesman heuristic. In *International Conference on Parallel Problem Solving from Nature*, pp. 95–107 (2018). 10.1007/978-3-319-99253-2_8.

[55] Burke, M. concorde TSP solver. https://github.com/matthelb/concorde (2015).

[56] Al-Omeer, M.A., Ahmed, Z.H. Comparative study of crossover operators for the mtsp. In *2019 International Conference on Computer and Information Sciences (ICCIS)*, pp. 1–6 (2019). 10.1109/ICCISci.2019.8716483.

[57] Duarte A, Pantrigo JJ, Pardo EG, Mladenovic N (2015). Multi-objective variable neighborhood search: An application to combinatorial optimization problems. J. Glob. Optim..

[58] Yang Y, Wu J, Sun X, Wu J, Zheng C (2013). A niched pareto tabu search for multi-objective optimal design of groundwater remediation systems. J. Hydrol..

[59] Jain, A. Local Search TSP. https://github.com/ayushjain1594/localsearchtsp (2020).

[60] Lust, T., Teghem, J. The multiobjective traveling salesman problem: A survey and a new approach. In *Advances in Multi-Objective Nature Inspired Computing*, pp. 119–141 (2010). 10.1007/978-3-642-11218-8_6.

[61] Paquete L, Stützle T (2009). Design and analysis of stochastic local search for the multiobjective traveling salesman problem. Comput. Oper. Res..

[62] Durillo JJ, Nebro AJ (2011). jmetal: A java framework for multi-objective optimization. Adv. Eng. Softw..

[63] Dexter F (2013). Wilcoxon–Mann–Whitney test used for data that are not normally distributed. Anesth. Analg..

[64] Nan, Z., Wang, X., Dib, O. Metaheuristic enhancement with identified elite genes by machine learning. In *International Symposium on Knowledge and Systems Sciences*, pp. 34–49 (2022). 10.1007/978-981-19-3610-4_3.

[65] Lust T, Jaszkiewicz A (2010). Speed-up techniques for solving large-scale biobjective tsp. Comput. Oper. Res..

